# Changes in social contact patterns in Germany during the SARS-CoV-2 pandemic – an analysis based on the COVIMOD study

**DOI:** 10.1186/s12879-025-10917-3

**Published:** 2025-04-23

**Authors:** Huynh Thi Phuong, Antonia Bartz, Andrzej K. Jarynowski, Berit Lange, Christopher I. Jarvis, Nicole Rübsamen, Rafael T. Mikolajczyk, Stefan Scholz, Tom Berger, Torben Heinsohn, Vitaly Belik, André Karch, Veronika K. Jaeger

**Affiliations:** 1 Institute of Epidemiology and Social Medicine, University of Münster, Münster, Germany; 2 System Modelling Group, Institute of Veterinary Epidemiology and Biostatistics, Freie Universität Berlin, Berlin, Germany; 3 Department of Epidemiology, Helmholtz Centre for Infection Research, Brunswick, Germany & German Centre for Infection Research, TI BBD, Brunswick, Germany; 4 London School of Hygiene and Tropical Medicine, London, UK; 5 Institute for Medical Epidemiology, Biometrics, and Informatics (IMEBI), Interdisciplinary Center for Health Sciences, Medical Faculty of the Martin Luther University Halle-Wittenberg, Halle, Germany; 6 Medical Faculty of the Martin Luther University Halle-Wittenberg, Halle, Germany, until 01/2022 Immunization Unit, Infectious Disease Epidemiology, Robert Koch-Institute, Berlin, Germany

**Keywords:** Contact behaviour, Heterogeneity, Modelling, Pandemic, SARS-CoV-2

## Abstract

**Background:**

During the SARS-CoV-2 pandemic, Germany employed several nonpharmaceutical interventions (NPIs) to reduce social contacts and decelerate the virus’s spread. Associations between demographics and other factors, e.g. perceived pandemic threat level, might help explain variations in social contact behaviours. We aimed to estimate contact numbers during the pandemic in Germany and assess factors associated with changes therein.

**Methods:**

Between 04/2020 and 12/2021, we conducted an online contact survey (COVIMOD) with 33 waves in Germany. We calculated the mean and 95% confidence interval of daily reported contacts (“people who you met in person and with whom you exchanged at least a few words, or with whom you had physical contact”) using bootstrapping. The effects of different factors on the number of contacts were determined by fitting generalized additive models (GAMs).

**Results:**

The COVIMOD survey recorded 59,585 responses from 7,851 participants across Germany. The overall mean number of daily social contacts during the study period was 3.30 (95%CI: 3.23–3.38), with the number of non-household contacts being twice as high as the number of contacts with household members. The lowest overall number of contacts (2.11, 95%CI: 2.01–2.22) was reported during Germany's strongest contact reduction campaigns (end of 04/2020), when the number of household contacts was three times higher than non-household contacts. The highest number of contacts (6.38, 95%CI: 5.67–7.15) was observed during periods of relaxed measures (June 2020), when household contacts were four times fewer than non-household contacts. The work and school contacts shaped the overall variation of contact patterns in Germany during the pandemic.

In participants under 18 years, partially/fully closing schools reduced school contacts by 83% (95%CI: 80–85%) and overall contacts by 39% (95%CI: 36–42%). Higher risk perceptions regarding COVID-19 were associated with 11% (95% CI: 2–17%) more social contacts among all participants and 66% (95%CI: 32–108%) more work contacts in the adult participants.

**Conclusions:**

Our study revealed fluctuations in the number of social contacts during the SARS-CoV-2 pandemic in Germany, with substantial variations influenced by NPIs and individual factors. Understanding these factors affecting social contacts is vital for refining disease transmission models and informing future pandemic response strategies.

**Supplementary Information:**

The online version contains supplementary material available at 10.1186/s12879-025-10917-3.

## Background

Social contact patterns play a fundamental role in the transmission of infectious diseases [1–4]. A good understanding of contact patterns and their heterogeneity among individuals is necessary to inform mathematical models assessing the effects of nonpharmaceutical interventions (NPIs) and shape future NPIs aiming to reduce contacts efficiently. During the COVID-19 pandemic, mathematical models have been pivotal in shaping NPI policies [3, 5]. However, these models often faced challenges due to insufficient data on how contact rates were affected, which could impact the effectiveness of policy measures [6, 7].

Several studies assessed contact patterns in various countries, e.g. the UK, the Netherlands, and Belgium [3, 8–14]. Using data from studies involved in or associated with the European-wide CoMix study [15], Wong et al*.* compared social contact patterns of 21 European countries during the pandemic and found considerably lower contact rates during the pandemic compared to before 2020, but also found differences in the reduction of contacts across the countries [16].

While other studies have examined changes in contact patterns as well as determinants of these changes during the SARS-CoV- 2 pandemic in various other countries, an in-depth analysis of this is missing for Germany [16]. Therefore, we aimed to analyse the contact patterns observed in the German COVIMOD study in a sample with a distribution representing the German population (with respect to age, sex, and federal states) through various phases of the SARS-CoV- 2 pandemic and to assess factors associated with the contact patterns. We present the results of 33 survey waves from the COVIMOD study from April 2020 through December 2021, encompassing times with strict contact reduction measures (April/May 2020 and winter 2020/2021), medium strict measures (autumn 2020 and spring and autumn 2021), and eased measures (summers 2020 and 2021). Specifically, we estimated contact frequency and duration (in hours), contact types, and contact settings throughout the pandemic and assessed factors influencing the contact numbers and duration within the specific context of Germany.

## Methods

### COVIMOD contact survey

The online contact survey COVIMOD was initiated in April 2020. We commissioned the market research company IPSOS-Mori to conduct the survey based on participants of the online-panel i-say.com [17]. Participants were recruited based on age, sex, and regional (federal states) quotas. Participants were asked to retrospectively report their social contacts of the previous day (between 5am the previous day to 5am on the day of the survey). A subgroup of adult participants with under-aged children living in their households was invited to provide information solely as a proxy for their children, allowing us to collect information on the social contacts of children (< 18 years). More details on COVIMOD can be found elsewhere [2, 18].

The timing and sample sizes of the 33 included COVIMOD survey waves (April 2020 to December 2021) can be found in Table 1. At the start of the study, 1560 participants were included and invited to participate longitudinally, however not all participants chose to do this. Therefore, at survey wave 4, an additional 1000 participants were invited to “boost” the sample size and were also invited to participate in the following survey waves. From wave 11 to wave 20 and from wave 21 onwards, the samples were “boosted” at each new wave to include a total of around 1500 and 2500 participants, respectively, i.e. for each survey wave, new participants were invited if the number of respondents was smaller than 1500 (waves 11–20) or 2500 (wave 21 onwards). The first survey wave corresponded to the time of the strictest contact reduction measures in Germany, whereas the other waves were rolled out at time points corresponding to different levels of contact reduction measures.

**Table 1:** Characteristics of participants and timeline of the COVIMOD contact survey in Germany, from April 2020 to December 2021. Across 33 survey waves, 0.3% of respondents responded with “In another way” or “Prefer not to answer” for sex and 0.1% with “Don’t know” and “Prefer not to answer” for age. Those were considered as missing

Wave	Number of participants	Timing	Sex (%)		Age in years (%)				Responded for weekday/weekend (%)		Household size
			**Female**	**Male**	**0–19**	**20–44**	**45–64**	**65 + **	**Weekend**	**Weekday**	**Median (IQR)**
1	1560	30.04.− 06.05.20	47.9	51.7	26.0	25.2	32.1	26.0	21.5	78.5	3 (2–4)
2	1356	14.05.− 21.05.20	47.1	52.9	26.8	24.5	33.4	26.8	13.6	86.4	3 (2–3)
3	1081	28.05.− 04.06.20	49.6	50.3	29.0	19.9	38.0	29.0	9.4	90.6	3 (2–3)
4	1890	11.06.− 22.06.20	47.7	52.1	25.9	26.0	31.5	25.9	16.6	83.4	3 (1–3)
5	1615	26.06.− 01.07.20	45.4	54.3	27.6	25.7	31.3	27.6	33.7	66.3	3 (2–3)
6	1496	09.07.− 16.07.20	46.5	53.3	25.0	25.7	32.6	25.0	16.6	83.4	3 (2–3)
7	1207	24.07.− 29.07.20	46.8	52.8	18.6	29.2	35.5	18.6	21.6	78.4	2 (2–3)
8	1022	07.08.− 11.08.20	46.7	53.2	23.4	27.7	33.5	23.4	22.9	77.1	2 (2–3)
9	869	04.09.− 09.09.20	44.5	55.4	27.0	29.6	26.6	27.0	25.3	74.7	3 (2–3)
10	739	30.09.− 05.10.20	42.6	57.4	32.2	25.8	28.0	32.2	0.8	99.2	2 (1–3)
11	1499	14.10.− 21.10.20	46.0	53.8	29.2	24.5	31.4	29.2	13.5	86.5	2 (1–3)
12	1500	29.10.− 03.11.20	46.0	53.9	30.5	24.4	29.7	30.5	10.7	89.3	2 (2–3)
13	1500	05.11.− 10.11.20	47.4	52.4	29.6	25.2	29.2	29.6	14.9	85.1	2 (1–3)
14	1490	25.11.− 30.11.20	47.1	53.0	25.4	27.5	30.6	25.4	15.8	84.2	2 (1–3)
15	1500	09.12.− 15.12.20	46.6	53.1	25.3	28.3	30.3	25.3	14.1	85.9	2 (1–3)
16	1499	23.12.− 30.12.20	47.2	52.5	25.2	28.8	30.2	25.2	7.8	92.2	2 (1–3)
17	997	28.01.− 02.02.21	46.3	53.5	24.8	29.0	29.6	24.8	1.2	98.8	2 (1–3)
18	1499	24.02.− 03.03.21	47.4	52.4	26.0	27.4	30.2	26.0	29.2	70.8	2 (1–3)
19	1498	17.03.− 26.03.21	47.1	52.7	25.2	28.7	29.6	25.2	19.4	80.6	2 (1–3)
20	1493	07.04.− 15.04.21	47.8	52.0	25.3	28.7	30.1	25.3	28.1	71.9	2 (1–3)
21	2468	12.05.− 24.05.21	47.2	52.5	25.4	26.9	30.3	25.4	15.4	84.6	2 (1–3)
22	2441	26.05.− 03.06.21	47.7	52.1	26.2	26.8	31.1	26.2	37.2	62.8	2 (1–3)
23	2485	09.06.− 22.06.21	47.0	52.7	26.0	27.1	30.7	26.0	28.6	71.4	2 (1–3)
24	2468	07.07.− 19.07.21	47.2	52.6	25.8	27.1	31.4	25.8	37.4	62.6	2 (1–3)
25	2480	04.08.− 13.08.21	47.4	52.3	25.6	27.8	31.8	25.6	31.3	68.7	2 (1–3)
26	2502	01.09.− 14.09.21	47.7	52.0	26.3	25.0	33.5	26.3	28.0	72.0	2 (1–3)
27	2492	22.09.− 06.10.21	46.1	53.7	29.2	19.9	35.7	29.2	9.2	90.8	2 (1–3)
28	2493	08.10.− 20.10.21	48.9	50.7	29.9	22.7	31.8	29.9	14.7	85.3	2 (1–3)
29	2487	22.10.− 02.11.21	48.9	50.7	26.1	24.1	32.5	26.1	27.8	72.2	2 (1–3)
30	2487	03.11.− 09.11.21	48.9	50.6	27.3	23.4	31.7	27.3	26.3	73.7	2 (1–3)
31	2489	17.11.− 23.11.21	48.1	51.7	26.7	23.0	33.1	26.7	13.8	86.2	2 (1–3)
32	2490	08–12.− 17.12.21	48.1	51.5	27.9	21.9	34.5	27.9	18.4	81.6	2 (1–3)
33	2493	24–12.− 31.12.21	48.7	51.0	27.0	24.8	31.6	27.0	30.4	69.6	2 (1–3)

The COVIMOD questionnaire is similar to the questionnaire used in the European-wide CoMix study, and includes questions on demographic characteristics, preventive behaviours, and risk perceptions regarding COVID-19, i.e."I am likely to catch coronavirus", "Coronavirus would be a serious illness for me", and "If I don't follow the government's advice, I might spread coronavirus to someone who is vulnerable” [18]. In addition, participants were asked to provide information about each of their social contacts with household members (household contacts) and with those outside of their household (non-household contacts) between 5 am the preceding day and 5 am on the day of the survey. This information included the age and sex of the contact, the time spent with each contact, and the setting where the contact occurred (i.e. at work, at school (including contacts at childcare, schools, and universities), at home, at somebody else’s home, at a place of worship, at a shop for non-essential items, at a place of entertainment (e.g. bar, restaurant, cinema), at a place of sport (e.g. gym, sports club), outside (e.g. at a park), at a beauty place (e.g. hairdresser, nail salon), while using transportation, in a healthcare setting (e.g. hospital, GP, dentist), at a shop for essential goods, and other settings).

In line with the contact definition used in POLYMOD (a landmark contact survey conducted from 2005–2006), a social contact in COVIMOD is defined as “people who you met in person and with whom you exchanged at least a few words, or with whom you had physical contact” [1]. During survey waves 1 and 2, participants were asked to provide all contacts separately. From wave 3 onwards, in addition to individual contacts, participants were given the opportunity to provide a number of contacts (from now on referred as “group contacts”) in each of three different settings (work, school, and other/not specified) and for three contact age groups in each setting (< 18 years, 18 to 64 years, 65 years or older) if the participant felt that they had too many contacts to report individually. The information obtained on these “group contacts” does not include information on the duration of the contacts or contact frequency.

The questionnaire can be found in Additional file 1.

### Information from secondary data sources

We extracted data on NPIs at the federal state levels from the healthcare data platform infas360 [19]. We encompassed only measures which might have potential implications for social contacts (including workplace restrictions and restrictions on leaving home). We gathered information on NPIs in nursery schools, schools, and universities by screening through each federal state’s press releases from ministry websites with the same method as used by Heinsohn et al. [20]*.* Data on school measures were collected on a weekly basis from 27 April 2020 to 2 January 2022 for all 16 federal states. School measures were categorised for an approximate percentage of attendance based on whether schools were completely or partially open (school attendance of more than 30%) vs. mostly closed (with emergency care for younger age groups) or during holidays (30% or less school attendance). Attendance was classified separately for nursery schools, primary schools, secondary schools, students in their final year of secondary school, and universities.

Social gathering measures were categorised into being in place or not based on workplace restrictions and stay-at-home orders (any restrictions on workplace attendance or leaving home vs. no restrictions in place).

In addition, the Stringency Index, provided by the Oxford COVID- 19 Government Response Tracker (OxCGRT), was used as a proxy for the strictness of nation-wide German government policies in response to the COVID- 19 pandemic [21]. The Stringency Index considers various policy indicators and tracks changes in government responses over time.

### Data management and analyses

We applied post stratification weights to the COVIMOD data based on the 2019 German projected census data according to the participant’s age, sex, household size, federal state, and day of the week (Additional file 2 Table AF13) using the R package “anesrake” [22, 23]. To prevent a few outliers with a large number of contacts from affecting the analyses, we truncated the number of additional work contacts, additional school contacts, and other additional contacts per participant at 100 contacts per setting in line with previous studies [2, 18].

Participants were asked how long they spent with the individual they had contact with. From survey waves 1 to 13, participants were allowed to specify the estimated time in hours and minutes. However, from wave 14 onwards, the question’s answers were reshaped to the category of 0–4 min, 5–14 min, 15–59 min, 1–4 h, and 4 h or more. To synchronise the data across the survey waves, we first converted contact time in waves 1 to 13 into the categories used in waves 14 to 33. Then, we randomly drew from a uniform distribution of the contact time range to derive individual assumed contact duration for all waves. The “4 h or more” range was set to a maximum of 16 h for household contacts and 8 h for non-household contacts.

We applied the bootstrapping method using the R package ‘boot’ with 1000 bootstrap samples [24, 25] to estimate the 95% confidence interval (CI) for the mean number of social contacts and the duration of contact (in hours) per survey wave. This analysis was conducted for all contacts and separately for household and non-household contacts. We further stratified non-household contacts by the setting where the contact happened.

We also assessed details of the non-household contacts, such as if the contact was a physical (e.g. hugging, kissing) or non-physical contact, if the contact took place inside or outside a building, the relationship between the participant and the contact person, and how often the participant and the contact were in contact prior to the pandemic. As this information was only available for the contacts recorded individually, i.e. not for the “group contacts”, the description of the contact details is restricted to the contacts recorded individually.

Finally, we investigated the effects of different potential determinants on social contacts using generalized additive models (GAMs), taking the longitudinal nature of the data into account [26]. We incorporated random effects for participants to model the variability within individuals over time and nested participants within federal states in Germany to account for regional variations. Additionally, spline terms were included to adjust for respondent fatigue (measured per participant as the n-th survey wave response) and for the effect of time (represented as year-month). We assumed the reported number of contacts followed a negative binomial distribution, which was modelled using a natural log link function.

The results (coefficients) from the GAMs were exponentially transformed and interpreted as Contact Number Ratio (CNR) or Contact Duration Ratio (CDR) for ease of interpretation. These ratios indicate the relative change in social contacts or contact duration associated with each determinant, quantifying the impact of various factors on social behaviours. Additionally, the percentage change can be calculated using the formula ((CNR or CDR) − 1) × 100%.

R version 4.3.2 [27] was used for all analyses.

## Results

### COVIMOD characteristics

Between April 2020 and December 2021, the COVIMOD study population was surveyed 33 times with a total of 739 to 2502 participants in each survey wave. In total, 59,585 responses by 7,851 individuals were recorded. Of the participants, 80% took part in two or more waves and 56% in more than five waves. Mean household size ranged from two to three and 28% of participants lived in a one-person household. Details of the demographic characteristics of the COVIMOD participants can be found in Table 1.

### Non-household versus household contacts

**Fig. 1:**
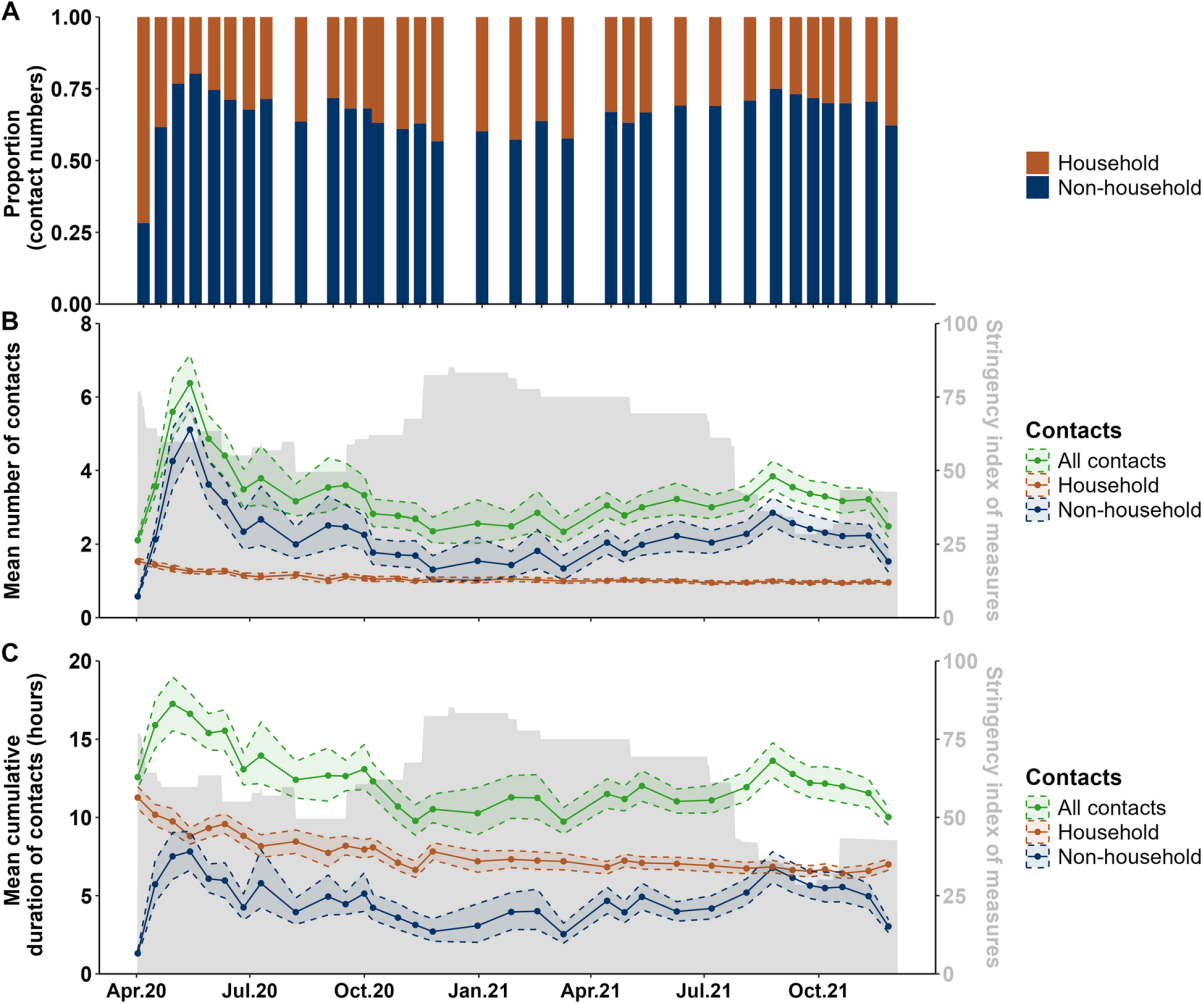
Social contact variation between April 2020 and December 2021 in Germany. **A** The proportion of household and non-household contacts among all contacts; (**B**) mean number of contacts per person per day; (**C**) mean cumulative duration of contacts per person per day (in hours) over time. For comparison, the stringency of the NPIs in Germany [21] over the study period is provided in grey shadow on the right y-axis for B and C. For B and C, the solid line represents the mean number of contacts; the shaded area illustrates the 95% CI obtained using bootstrapping

In the first survey wave, only 28% of all social contacts were with a person not belonging to the household of the surveyed person (non-household contacts; Fig. 1A). This proportion increased to 80% in June 2020 and stabilised between 58–74% for the rest of the survey waves. As expected, the mean daily social contact numbers within a household remained stable between 1 and 1.5 during the study period. In contrast, the non-household contacts varied over time and shaped the overall social contact trends (Fig. 1B).

### Number and duration of social contacts over time

On average, a participant had 3.30 (95% CI: 3.23–3.38) contacts per day, of which 1.06 (95% CI: 1.05–1.07) contacts were with household members and 2.24 (95% CI: 2.17–2.32) contacts were with non-household members (Additional file 2 Table AF1). However, the average number of contacts varied heavily over the study period. During Germany's strongest contact reduction measures (end of April 2020), the mean number of contacts per day was reduced to 2.11 (95% CI: 2.01–2.22). This number increased to between 4.40 (95% CI: 3.80–5.01) and 6.38 (95% CI: 5.67–7.15) contacts per day during May and mid July 2020 when the restrictions were relaxed. After that, the mean number of daily social contacts stabilised between 3.16 (95% CI: 2.76–3.63) and 3.79 (95% CI: 3.06–4.67). At the end of October 2020, the social contacts continued going down to between 2.35 (95% CI: 2.03–2.73) and 2.85 (95% CI: 2.36–3.43), which was in line with the nationwide “lockdown light” imposed by the German government in early November 2020 till the end of April 2021. Later, social contact numbers rose again at the start of the new school year in September 2021, when the contact reduction measures were at the lowest stringency. Consequently, the daily social contacts went up to 3.84 (95% CI: 3.48–4.26) in early October 2021 (Fig. 1B, Additional file 2 Table AF1).

When analysing the duration of contacts, an individual's total duration (in hours) of contacts per day showed a similar pattern as the number of contacts, though the magnitude of variation was smaller (Fig. 1C). On average, individuals spent roughly 12.28 h (95% CI: 12.08–12.47) per day in contact with someone else (Fig. 1C, Additional file 2 Table AF1). In contrast to contact numbers, an individual spent more time per day in contact with household members (7.52 h (95% CI: 7.43–7.60)) than with those not belonging to their household (4.76 h (95% CI: 4.59–4.93)) (Fig. 1C, Additional file 2 Table AF1).

We observed notable differences in social contact patterns when stratifying all contacts by age group. Individuals aged 0–19 consistently reported the highest mean number of contacts, ranging from 3.03 (95% CI: 2.73–3.35) to 9.72 (95% CI: 7.93–11.68) during the study period, while the mean number of contacts in those aged 20–49 ranged from a minimum of 1.81 (95% CI: 1.61–1.99) to a maximum of 7.26 (95% CI: 5.69–9.00) during the survey waves (Figure AF3 A, Table AF15). The 50–69 age group exhibited lower contact numbers with 1.83 (95% CI: 1.71–1.97) to 4.94 (95% CI: 3.94–6.07) mean contacts per day, while the 70 and older group had the fewest contacts, ranging from 1.64 (95% CI: 1.39–1.90) to 4.08 (95% CI: 2.78–5.65) mean daily contacts. Furthermore, younger age groups (0–19) tended to report longer durations of contact (average 23.36 h (95% CI: 22.71–23.99) compared to older groups (around 10 h) (Figure AF3B, Table AF15).

### Factors associated with contacts in different settings over time

**Fig. 2:**
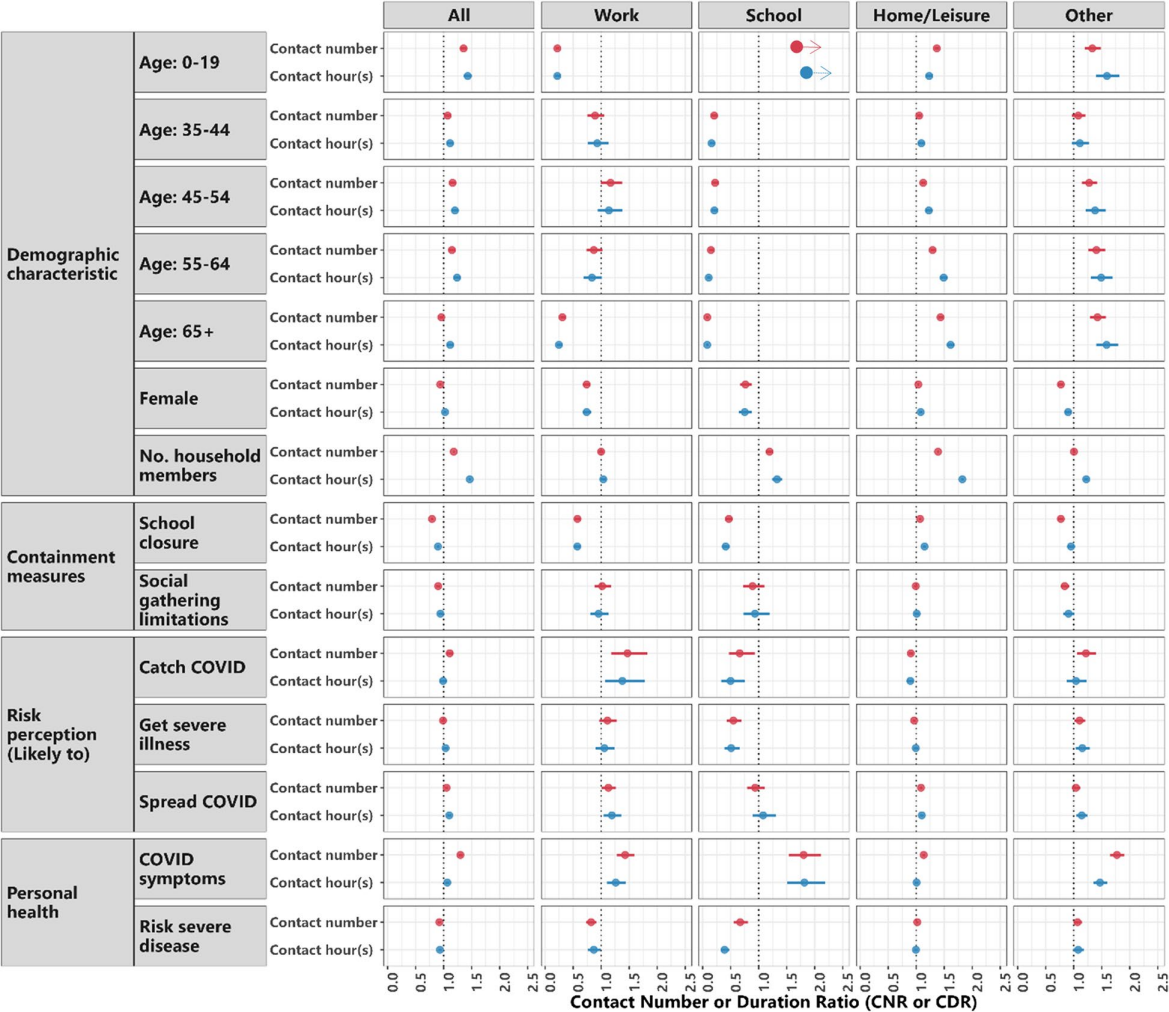
Factors associated with contacts by settings. Depicted are the Contact Number Ratio (CNR) or Contact Duration Ratio (CDR) among individuals at a specified setting, including all settings, work, school, home/leisure, and other settings. Dots represent the point estimates from GAM models; horizontal lines represent the 95% CI. These estimates were derived from multivariable analysis. The reference groups for comparison are 20-34 for age groups; male for sex; less than 30% closing for school closure; no measures in place for social gathering limitations (e.g. no workplace restriction or requiring a stay-at-home); “strongly agree” compared to a less strongly opinionated group (which includes "strongly disagree", "tend to disagree", "neutral", and "tend to agree") for risk perceptions; no COVID-19 symptoms and no risk for severe disease if contracted COVID-19 for personal health. For the grouping of the contact settings into home/leisure and other, please look at Additional file 2 Table AF14. The points with arrows presesent the school estimates for age 0-19 are 9.58 (95% CI 7.91-11.61) for the contact numbers and 13.19 (95% CI 10.35-16.82) for contact duration (hours)

Females reported 6% (95% CI: 4–8%) less contacts than males (Fig. 2, Additional file 2 Table AF2). This was more pronounced for work contacts among participants aged over 18 with females reporting 13% (95% CI: 5–21%; Additional file 2 Figure AF2, Additional file 2 Table AF4) fewer contacts. Meanwhile, females showed 4% (95% CI: 2–6%) more home and leisure-time contacts than males.

Compared to the reference age group of 20–34-year-olds, those under 20 and aged 35–64 reported 36% (95% CI: 30–42%) and 7% to 16% (95%CI: 2–21%), respectively, more contacts overall (Fig. 2, Additional file 2 Table AF2). Among participants aged over 18, those aged 35–44 and 55–64 reported little to no difference in work contacts but those aged over 64 showed 42% (95% CI: 29–58%) more contacts in other settings compared to 20–34-year-olds.

During times when schools were partially or fully closed, there was a reduction in contacts at schools by 53% (95% CI: 46–59%), contacts at work by 42% (95% CI: 37–48%), and contacts at other settings by 23% (95% CI: 18–28%), but an increase in contacts at home or leisure places by 7% (95% CI: 5–9%) among all participants (Fig. 2, Additional file 2 Table AF2). These effects were primarily visible among children (18 years old or under), where contacts at schools were reduced by 83% (95% CI: 80–85%) and overall contacts by 39% (95% CI: 36–42%) (Additional file 2 Figure AF1, Additional file 2 Table AF3). Among all participants, workplace restrictions or stay-at-home orders (social gathering limitations) had no large impact on contacts at work or school, but still reduced overall contact numbers by 10% (95% CI: 6–13%) (Fig. 2, Additional file 2 Table AF2). If restricting the study population to those aged over 18, social gathering limitations led to a reduction of 21% (95% CI: 8–32%) in contacts at work and 11% (95% CI: 7–15%) in contacts overall (Additional file 2 Figure AF2, Additional file 2 Table AF4).

When restricting the populations to the adult participants (over 18 years), those who perceived their risk of getting a COVID-19 infection as very high reported 66% (95% CI: 32–108%) more contacts at work (Additional file 2 Figure AF2, Additional file 2 Table AF4). Among the adult participants, those who thought that they would be likely to experience a severe course of COVID-19 reported 17% (95% CI: 1–35%) more contacts at work, and those worried about spreading SARS-CoV-2 to vulnerable persons reported 9% (95% CI: 6–12%) more contacts at home or in leisure places and 7% (95% CI: 3–10%) more contacts overall (Additional file 2 Figure AF2, Additional file 2 Table AF4).

### Contacts by settings

**Fig. 3:**
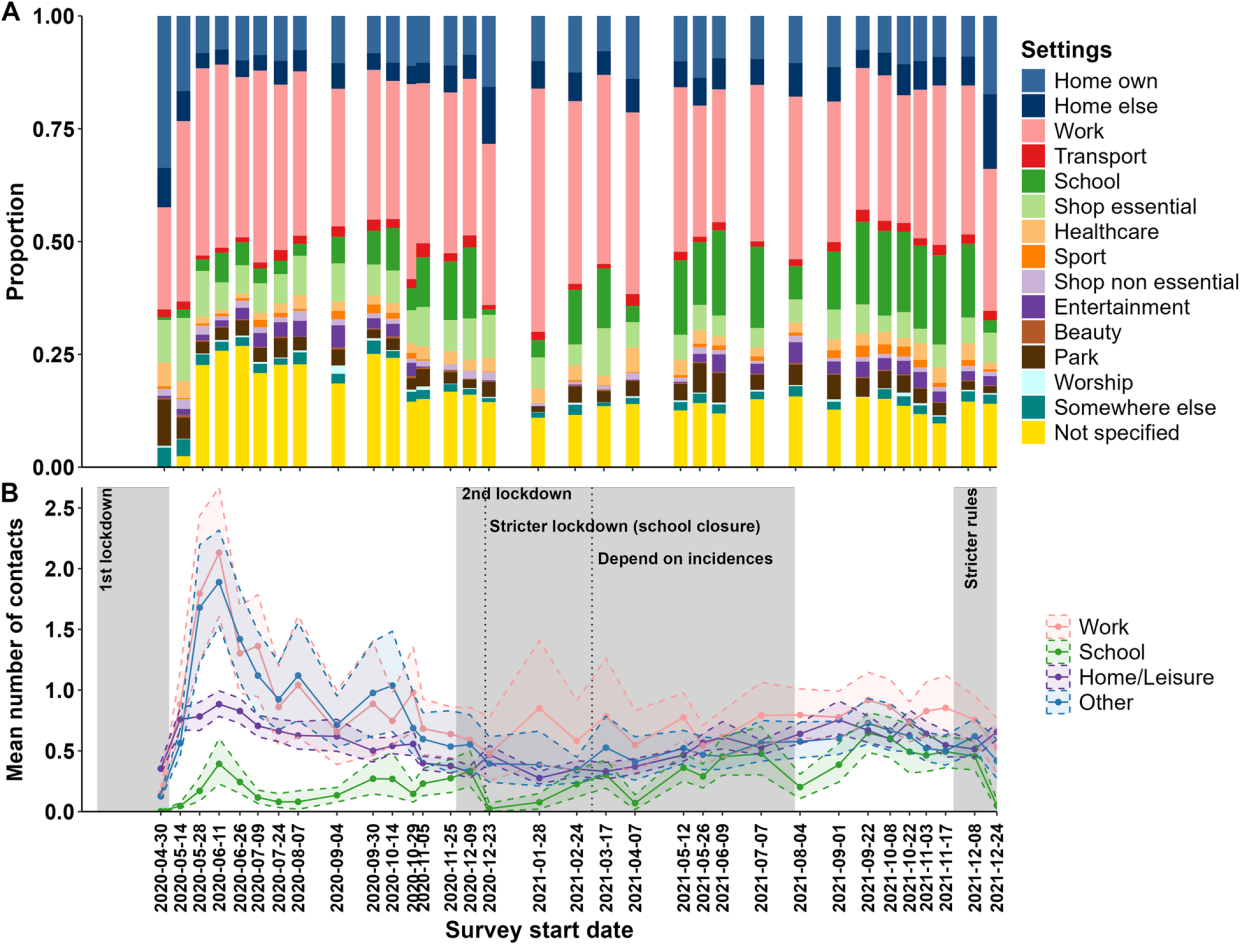
Settings of non-household contacts between April 2020 and December 2021 (including group contacts). **A** Distribution of non-household contacts in different settings. **B** Setting-specific means of non-household contact numbers stratified by work contacts, school contacts, home/leisure contacts, and contacts in other settings. For the grouping of the contact settings into home/leisure and other, please look at Additional file 2 Table AF14. For plot A, the “not specified” category includes contact situations where no specific setting was reported, and all “group” contacts were reported at “other/not specified settings”. For plot B, the solid line represents the mean number of contacts; the shaded area is the 95% CI obtained using bootstrapping. The grey areas indicate when stricter containment measures were in place (OxGCRT stringency index ≥ 67)

On average, 35% of non-household contacts occurred at work, 11% at school, 25% at leisure locations, and 29% at other locations (Fig. 3A, Additional file 2 Table AF5).

As shown in Fig. 3B, the mean number of contacts at work and school substantially changed over time and fluctuated strongly depending on the restriction measures. In contrast, contacts taking place at home or at leisure places were rather stable. The contacts at other settings varied from the start of the pandemic till the end of 2020 and became more stable in 2021.

### Contacts by physical proximity

**Fig. 4:**
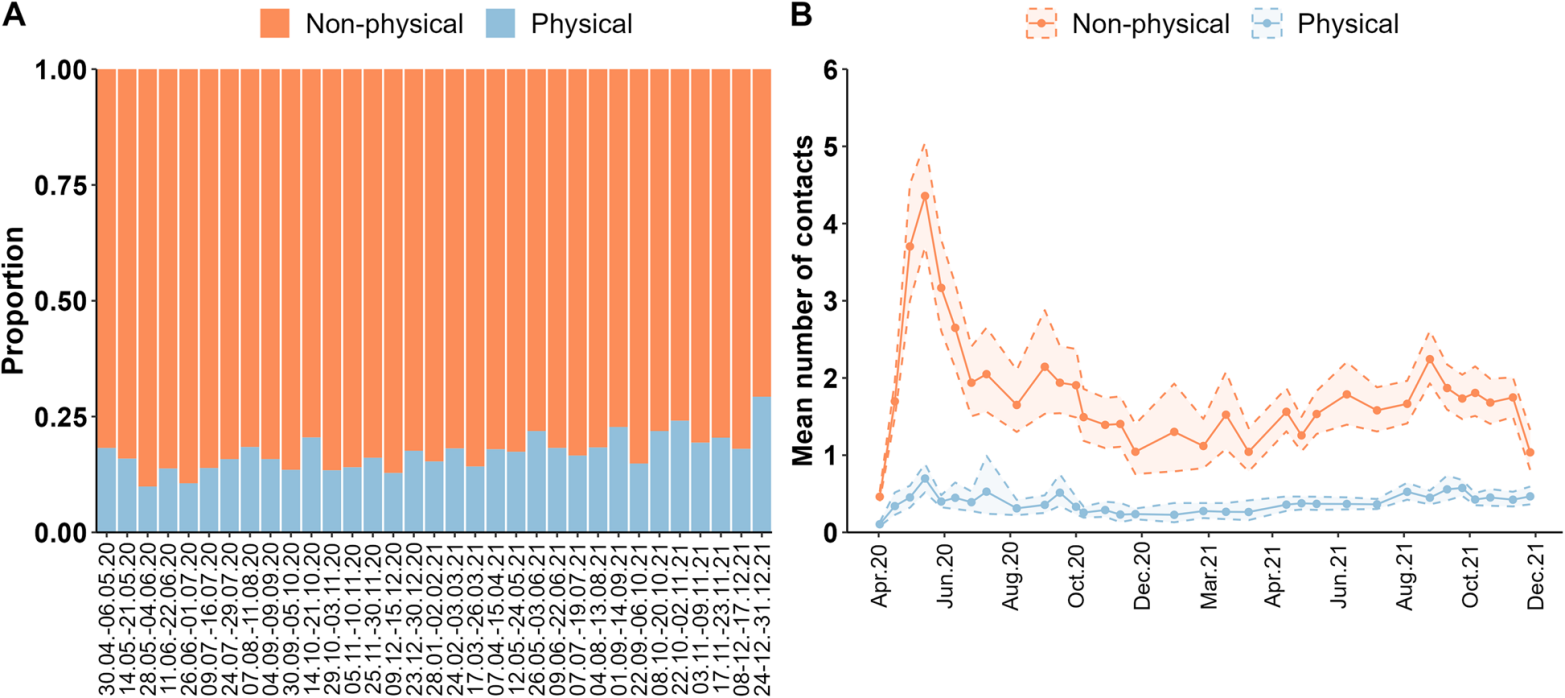
Contact types (physical (e.g. handshaking, hugging, kissing) and non-physical) of non-household contacts (including group contacts). **A** Distribution of contact types of non-household contacts per survey wave; (**B**) mean number of non-household contacts stratified by contact type over time by survey wave

Approximately 10–29% of non-household contacts were physical (Fig. 4A, Additional file 2 Table AF6). The daily number of physical non-household contacts ranged between mean values of 0.11 (95% CI: 0.08–0.13) and 0.70 (95% CI: 0.52–0.89); the mean number of non-physical contacts varied much more than physical contacts, ranging from 0.46 (95% CI: 0.40–0.53) to 4.36 (95% CI: 3.70–5.05; Fig. 4B, Additional file 2 Table AF6).

**Fig. 5:**
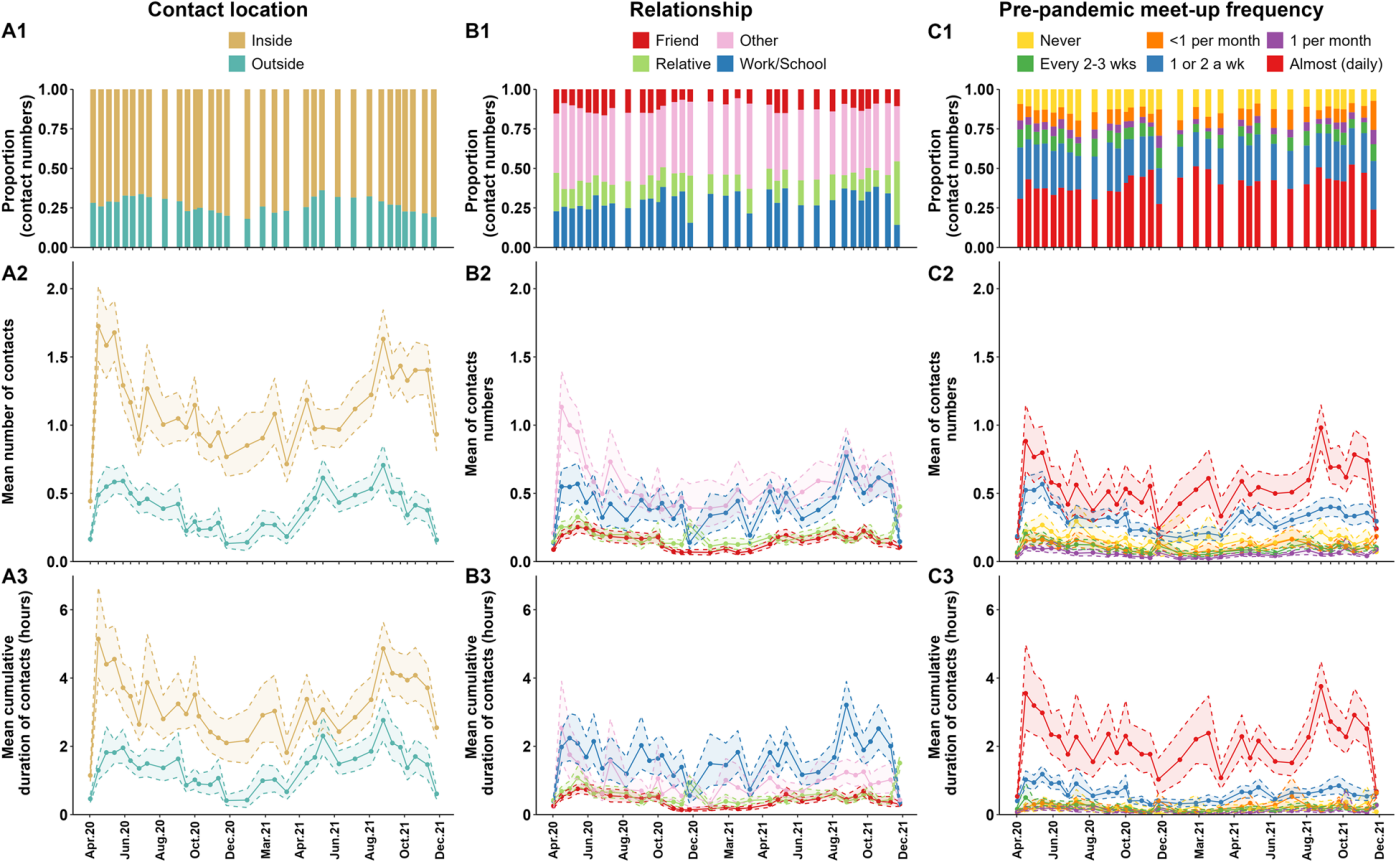
Contact location, relationship, and pre-pandemic meet-up frequency of non-household contacts (individual contacts only). (A1, B1, C1) Distribution of contacts; (A2, B2, C2) mean numbers of contacts; and (A3, B3, C3) mean duration of contacts (in hours) by contact location (A), relationship (B) and pre-pandemic meet-up frequency (C). The solid line represents the mean number of contacts; the shaded area is the 95% CI obtained using bootstrapping

### Contacts by location (inside a building vs. outside)

The majority of recorded non-household contacts took place inside (62–87%; Fig. 5 A1, Additional file 2 Table AF7). Participants reported a mean of 1.15 (95% CI: 1.12–1.18) inside and 0.40 (95% CI: 0.39–0.42) outside contacts (Fig. 5 A2, Additional file 2 Table AF7). A non-household contact inside a building lasted on average 3.28 h (95% CI: 3.15–3.42) and a contact taking place outside lasted on average 1.46 h (95% CI:1.38–1.53; Fig. 5 A3, Additional file 2 Table AF7). Across survey waves, participants reported 2 to 6 times more non-household contacts taking place inside than outside; similarly, the duration of the contacts were 1.4 to 4.5 times longer with contacts taking place inside compared to outside. As expected, more contacts took place outside during the summer months than during the winter months (Fig. 5 A2 and 5 A3, Additional file 2 Table AF7).

### Contacts by relationship with contact persons

Overall, 31% of non-household contacts were with someone from work or school, 14% with relatives, 12% with friends, and 43% with other persons such as clients and babysitters (Fig. 5B1, Additional file 2 Table AF8). Contacts with relatives remained stable during the survey period, averaging 0.19 contacts per day (95% CI: 0.19–0.20) and 0.57 h of interaction per day (95% CI: 0.55–0.60); however, these figures rose to 0.40 contacts (95% CI: 0.35–0.45) and 1.51 h (95% CI: 1.31–1.71) during Christmas time in 2021 (Fig. 5B2 and 5B3, Additional file 2 Table AF8, Additional file 2 Table AF9). Work or school contacts ranged between 0.14 (95% CI: 0.09–0.19) and 0.78 (95% CI: 0.65–0.91) contacts daily and 0.35 (95% CI: 0.24–0.50) to 3.21 (95% CI: 2.57–3.90) hours. Interaction with friends was consistent, averaging 0.16 contacts (95% CI: 0.15–0.16) and 0.44 h (95% CI: 0.42–0.46) daily. Contacts that fell into the “other” category had the least stable pattern, with 0.21 (95% CI: 0.18–0.25) to 1.13 (95% CI: 0.92–1.39) contacts and 0.23 (95% CI: 0.18–0.29) to 2.54 h (95% CI: 1.55–3.90) daily (Figs. 5B2 and 5B3; Additional file 2 Tables AF8 and AF9).

### Contacts by frequency of meeting the contact person before the pandemic

Most reported non-household contacts (65%) were with persons with whom participants reported having had regular contact before the pandemic, such as at least once or twice a week (25%) or almost every day (40%), while only 12% of contacts were with someone never met before (Fig. 5 C1, Additional file 2 Table AF10). Across the COVIMOD study period, the mean number of non-household contacts ranged from a minimum of 0.17 (95% CI: 0.14–0.21) to a maximum of 0.98 (95% CI: 0.83–1.15) for those contacts who met almost daily and from a minimum of 0.17 (95% CI: 0.14–0.21) to a maximum of 0.57 (95% CI: 0.49–0.66) for those who met once or twice a week (Fig. 5 C2, Additional file 2 Table AF11). However, the mean number of non-household contacts was much lower for those contacts who met less frequently before the pandemic (i.e. never, less than once a month, once a month, or every two to three weeks), ranging from a minimum of 0.02 (95% CI: 0.01–0.04) to a maximum of 0.30 (95% CI: 0.19–0.42) during the study period (Fig. 5 C2, Additional file 2 Table AF11).

Similarly, individuals spent more time per day with those they met almost daily before the pandemic (minimum of 0.53 (95% CI: 0.43–0.65) to a maximum of 3.76 h (95% CI: 3.03–4.49) over the study period) compared to those who they met less than once per week before the pandemic (0.12 (95% CI: 0.11–0.14) to 0.64 (95% CI: 0.49–0.79) hours per day; Fig. 5 C3, Additional file 2 Table AF12).

## Discussion

The COVIMOD survey repeatedly collected social contact data from a representative sample in Germany during two years of the COVID- 19 pandemic, with a total of 59,585 survey responses in 33 surveys. The mean contact rate fluctuated over the study period depending on different factors. However, it remained lower than the pre-pandemic level of 7.95 (SD:6.26) contacts per day [1]. It is important to note that this pre-pandemic estimate, originating from data collected in 2005/2006, may not accurately reflect social behaviour just prior to the pandemic due to changes in demographics and social norms over time. Despite these uncertainties, the comparison suggests the impact of the pandemic on social interactions.

The differences in contact patterns across social groups can shape the spread of infectious diseases and the effectiveness of different non-pharmaceutical interventions [28]. Großmann et al*.* showed the importance of adequately capturing the heterogeneity of social contacts when modelling an infectious disease [29]. The COVIMOD study allowed us to characterise differences in contact patterns between different demographic groups in Germany during the pandemic for the first time. These findings can help researchers incorporate detailed parameters and boundary conditions in the modelling process. By integrating these factors, predictions of the spread of infectious diseases can be refined, allowing for more accurate results.

Prior research has consistently highlighted the substantial impact that school closures can have on reducing social contact during pandemics. Studies have shown that the necessity to restrict contacts often leads to the implementation of contact reduction measures such as school closures, which decrease contact rates within educational settings and influence interactions in other environments by reducing overall mobility and prompting stricter adherence to other containment measures [30, 31]. Additionally, implementing school closures often coincides with enforcing other stringent interventions, amplifying their collective effect on curbing virus transmission [32, 33]. The psychological signal accompanying school closures can further enhance public compliance with recommended behaviours [34].

Aligned with these findings, our research demonstrated that containment strategies, including the closure of schools and the stay-at-home orders, reduced the frequency of social contacts. The findings indicated that stricter measures led to notably fewer interactions among individuals. This reduction in contact numbers became increasingly apparent as the pandemic progressed, rather than in its initial stages. This suggests a growing level of caution and an adaptation in social distancing practices among the general population as they adjusted to the prolonged nature of the pandemic.

The spike in contacts during June 2020 is noteworthy. A similar increase in contacts was also observed in other European countries during the summer of 2020 [16]. Although it could be attributed to several factors, including a temporary relaxation of restrictions specific to this phase of the pandemic, contact numbers did not reach that peak again from July 2020 to December 2021. This indicates a unique phenomenon during June 2020, possibly influenced by specific contextual factors, that warrants further investigation.

Our study had some limitations. First, COVIMOD covered a wide temporal range (nearly 21 months), which includes the seasonal effects on social contacts. For instance, more social activities in the summer or end-of-year holidays were expected to be associated with more contacts. Second, we observed survey fatigue in our study. Participants tended to report fewer contacts the longer they participated in our study. Third, the online nature of the survey may have introduced potential biases, such as sample selection bias and underrepresentation of individuals with limited internet access. Additionally, the nature of self-reporting may have impacted the accuracy and completeness of contact data. Future studies could address these challenges by integrating newer methods, such as real-time digital tracking through mobile apps and wearable devices, which provide continuous and objective data on social contacts. Combining these technologies with traditional survey methods could help mitigate issues like survey fatigue and recall bias in reporting.

## Conclusion

Our study, conducted from April 2020 to December 2021, revealed insights into the dynamics of social contacts during the SARS-CoV-2 pandemic in Germany. We observed fluctuations in daily contact rates, with substantial variations influenced by containment measures and individual factors. Factors such as sex, age, household size, and risk perceptions of COVID-19 played important roles in shaping contact patterns in different settings. NPIs, particularly during periods of restrictions when school closures were in place, had a profound impact on reducing social contacts, thereby underlining their critical role in controlling the spread of the virus. Understanding the diverse factors that influence social contact behaviour is essential for enhancing disease transmission models and informing and guiding future pandemic response strategies. This underscores the importance for designing interventions specifically tailored to address distinct demographic and behavioural aspects, ensuring that response strategies are both effective and contextually appropriate. These insights might help to facilitate a more nuanced understanding of the impact of public health policies and help in crafting strategies that can be more responsive and adaptable to impact fast evolving epidemic situations.

## Supplementary Information

Additional file 1.

Additional file 2.

## Data Availability

The datasets used and/or analysed during the current study are available from the corresponding author on reasonable request.

## Abbreviations

CDR: Contact Duration Ratio
CNR: Contact Number Ratio
GAMs: Generallized addictive models
COVID- 19: Coronavirus disease 2019
NPIs: Non-pharmaceutical interventions
OxCGRT: Oxford COVID-19 Government Response Tracker
SARS-CoV- 2: Severe acute respiratory syndrome coronavirus 2
