# Supplementary material for: Changes in social contact patterns in Germany during the SARS-CoV-2 pandemic – an analysis based on the COVIMOD study
Source: BMC Infect Dis. 2025 Apr 23;25:588. doi: 10.1186/s12879-025-10917-3 (PMC12020284; doi:10.1186/s12879-025-10917-3)
Supplement: Supplementary file 1 — Additional file 1. [file 12879_2025_10917_MOESM1_ESM.pdf]

Changes in social contact patterns in Germany during the SARS-CoV-2 pandemic – an analysis based on the COVIMOD study.

Additional File 1: COVIMOD Questionnaire relevant to this study

Huynh Thi Phuong[1], Andrzej K. Jarynowski[2], Antonia Bartz[1], Berit Lange[3], Christopher I. Jarvis[4], Nicole Rübsamen[1], Rafael T Mikolajczyk[5], Stefan Scholz[6], Tom Berger[1], Torben Heinsohn[3], Vitaly Belik[2], André Karch[1], Veronika K Jaeger[1]

1 Institute of Epidemiology and Social Medicine, University of Münster, Münster, Germany

2 System Modelling Group, Institute of Veterinary Epidemiology and Biostatistics, Freie Universität Berlin, Germany

3 Department of Epidemiology, Helmholtz Centre for Infection Research, Braunschweig, Germany & German Centre for Infection Research, TI BBD, Braunschweig, Germany

4 London School of Hygiene and Tropical Medicine, London, UK

5 Institute for Medical Epidemiology, Biometrics, and Informatics (IMEBI), Interdisciplinary Center for Health Sciences, Medical Faculty of the Martin Luther University Halle-Wittenberg, Halle, Germany

6 Medical Faculty of the Martin Luther University Halle-Wittenberg, Halle, Germany, until 01/2022 Immunization Unit, Infectious disease epidemiology, Robert Koch-Institute, Berlin, Germany

Corresponding author:

Veronika K Jaeger, PhD

Institute of Epidemiology and Social Medicine

University of Münster

48149 Münster

Germany

Email: veronika.jaeger@ukmuenster.de

Keywords

Contact behaviour, heterogeneity, modelling, pandemic, SARS-CoV-2

Demographics

Q1. What is your age in years? YEAR/MONTH.

Q2. Which of the following describes how you think of yourself?

Male
Female
In another way
Prefer not to answer

Q20. Not including you, how many other people live in your household? By household, we mean anyone living at the same address as you, that you share a kitchen with.

None
1
2
3
4
5
6
7
8
9
10
11 or more

Q21. Please write the nickname of each other person in your household.
Note that this nickname is only needed to make it easier for you to complete the survey, so please pick a nickname that will help you identify each household member later in the questionnaire. Nicknames are not visible to anyone outside of this survey.

NAME 1
NAME 2
NAME 3
………..

Q23. Which of the following age groups do they fit into?

Under 1
1-4
5-9
10-14
15-19
20-24
25-34
35-44
45-54
55-64
65-69
70-74

75-79
80-84
85 years or older Don’t know
Prefer not to answer

Q24. As far as you know, which of the following describes how [NAME] think of themselves?
Male
Female
In another way
Prefer not to answer
Don’t know

Q28a. Are you or any other household member in in a high-risk group, meaning you/they could have serious symptoms if you contracted Coronavirus (COVID-19)?
High risk groups include individuals who: have had an organ transplant, undergoing cancer treatment, have blood or bone marrow cancer, have had a bone marrow or stem cell transplant in the past 6 months, are taking immunosuppressant medicine or high doses of steroids, have a severe lung condition (such as cystic fibrosis, severe asthma or severe COPD), have a condition that makes risk of getting infections higher (e.g. SCID or sickle cell), and/or are pregnant and have a serious heart condition

ROWS:
0. Yourself
1. Name 1
2. Name 2 etc.

COLUMNS:
1. Yes
2. No
3. Don’t know
4. Prefer not to answer

Symptoms

Q29. Have you, or anyone else in your household, had any of the following symptoms in the last seven days?

ROWS:
0. Yourself
1. Name 1
2. Name 2 etc.

COLUMNS:
1. Fever or high temperature
2. A cough that has lasted for at least several hours
3. Shortness of breath
4. Aches and pains, e.g. in back, neck, shoulders or joints
5. Blocked nose
6. Sore throat
7. Feeling unusually tired
8. None of these
9. Don’t know

10. Prefer not to answer

Attitudes

Q35. To what extent do you agree or disagree with each of the following statements?

Strongly agree
Tend to agree
Neither agree nor disagree
Tend to disagree
Strongly disagree
Don't know

Statements:

1. Coronavirus would be a serious illness for me
2. I am likely to catch coronavirus
3. If I don't follow the government's advice, I might spread coronavirus to someone who is vulnerable

Contact survey

We will now ask you to remember who [you have /NAME has] been in contact with yesterday, between 5am yesterday and 5am today.

These questions are voluntary, but they are really important in helping us understand the spread of COVID-19 and the impact of different public health interventions. It will not be possible to identify you or any member of your household in the published findings.
We are only interested in direct contacts, which are people who [you/NAME] met in person and with whom [you/NAME] exchanged at least a few words, or with whom [you/NAME] had physical contact (e.g. a handshake, embracing, kissing, contact sports).
Note that if [you/NAME] only spoke to someone over the phone or internet, they should not be included in this section.

Q62. Which of the following people did [you/NAME] have direct contact with in person, between 5am yesterday and 5am today, in person?

We are only interested in direct contact, which are people who you met in person and with whom you exchanged at least a few words, or with whom you had physical contact (e.g. a handshake, embracing, kissing, contact sports).
Note that if you only speak to someone over the phone or internet, they should not be included in this section.

COLUMNS:

1. Yes
2. No

CONTACT2

Q63. And what other people, outside of your household, did [you/NAME] have direct contact with in person, between 5am yesterday and 5am today? This could include friends, family, work colleagues, or people [you/NAME] spoke to in shops and so on.

Please write the nickname of each person [you/NAME] had direct contact with below. Note that this nickname is only needed to make it easier for you to complete the survey, so please pick a nickname that is easy to remember. Your individual response will not be shared with anyone outside of this survey.
We are only interested in people who you met in person and with whom [you/NAME] exchanged at least a few words, or with whom [you/NAME] had physical contact (e.g. a handshake, embracing, contact sports).

It is easiest list names in chronological order, e.g. After [I/NAME] had breakfast at home, [I/NAME] went to work where [I/NAME] met with Jack, Deborah, and two clients. On the way back home, [I/NAME] chatted with the shop assistant at the petrol station (give a nickname like "shop assistant"). When [I/NAME] returned home, [I/NAME] accepted a package from the delivery person, and [I/NAME] spoke to a friend, Fatima, in the garden. Etc.

Please do not list yourself, anyone that you listed as being a part of your household, or anyone who you only spoke to over the phone or internet.
PN: SHOW IFQ62 for any coded 1 You have already indicated contact with the following household members, add additional contacts in the textboxes below:

Male
Female
In another way
Prefer not to answer
Don't know

Q68. What is [NAME]'s relationship to [you/NAME]?

1. They are a family member who is not in my household 2. They are someone [I work/NAME works] with
3.They are someone [I go to school, college or university with/NAME goes to school, college or university with]
4.a boyfriend/girlfriend
5.other
6.I do not want to give any information

Q69. Before the coronavirus epidemic started, how often did [you/NAME] usually have direct contact with [NAME]? A direct contact is when you meet with this person in person and when you exchange at least a few words, or when you have physical contact (e.g. handshake, embracing, kissing, contact sports).
Please do not include times that you speak to them over the phone or internet.

1. Every day or almost every day
2. About once or twice a week
3. Every 2-3 weeks
4. About once per month
5. Less often than once per month

6. Never met them before
7. Prefer not to answer

Q70. When [you/NAME] had/had an immediate contact with [NAME] yesterday, [you/NAME] had/had a

1.physical contact (any kind of skin contact) (such as shaking hands, hugging, or kissing)?
2.non-physical contact (you did not touch the person)?
3.I do not want to give any information on this

Q71. And where did [you/NAME] have direct contact with [NAME]?

1. At home (including at your door, in your garden, and within entrances to your home such as stairways, lifts, and corridors)
2. At someone else's house
3. At work
4. At a place of worship
5. On any form of transport
6. At university, school, pre-school, or nursery
7. At a shop for essentials, eg a supermarket, grocery store, market, pharmacist, or bicycle shop
8. At a shop for non-essential items, eg a gardening center, or a clothes, electronics, furniture, or DIY shop
9. At a place of entertainment such as a restaurant, bar, cinema
10. At a place for sports such as a gym or sports club/match [PN. DO NOT SHOW IN BE AND NL]
11. Outside, for example in a park, on the street or in the countryside
12. In a healthcare setting, eg hospital, GP, A&E, outpatient facility, dentist, physiotherapist, optometrist, etc
13. At a hair dresser, barber, nail salon, beauty parlor or similar location
14. Somewhere else (please specify)

Q72. Please estimate the total amount of time [you/NAME] spent with [NAME] in person yesterday.

Q73. Was the time [you/NAME] spent with [NAME] yesterday inside or outside? Please tick all that apply
Inside
Outside

Q74. We do ask you to individually include every contact you had, but if you were unable to include every single contact (for instance, because you work in a shop and have a large number of contacts in a day), please could you indicate this?

1. I individually included every person I had contact with.
2. I did not individually include every person I had contact with.
3. I did not have any contacts

Q75. Approximately how many people have you had contact with that you did not specify individually? Please provide as accurate an estimate as possible, including age and

environment.
For the purpose of this question, we are only interested in people with whom there was direct contact and with whom you exchanged at least a few words OR with whom you had physical contact (e.g., shaking hands, hugging, contact sports).
Please indicate the number of contacts for each age group and setting.

ROWS
1. Under 18 years
2. 18 to 64 years
3. 65 years and older

COLUMNS
1. At the workplace
2. At school or other educational institution
3. Elsewhere

Q76. And approximately how many people did you have physical contact with that you did not specify individually? Please provide as accurate an estimate as possible, including age and environment. For the purpose of this question, we are only interested in people with whom therefore direct contact AND with whom you had physical contact (e.g., shaking hands, hugging, playing contact sports).
Please indicate the number of contacts for each age group and setting.

ROWS
1. Under 18 years
2. 18 to 64 years
3. 65 years and older

COLUMNS
1. At the workplace
2. At school or other educational institution
3. Elsewhere
