# Supplementary material for: Changes in social contact patterns in Germany during the SARS-CoV-2 pandemic – an analysis based on the COVIMOD study
Source: BMC Infect Dis. 2025 Apr 23;25:588. doi: 10.1186/s12879-025-10917-3 (PMC12020284; doi:10.1186/s12879-025-10917-3)
Supplement: Supplementary file 2 — Additional file 2. [file 12879_2025_10917_MOESM2_ESM.pdf]

Changes in social contact patterns in Germany during the SARS-CoV-2 pandemic – an analysis based on the COVIMOD study.

Additional File 2: Additional information and results

Huynh Thi Phuong[1], Andrzej K. Jarynowski[2], Antonia Bartz[1], Berit Lange[3], Christopher I. Jarvis[4], Nicole Rübsamen[1], Rafael T Mikolajczyk[5], Stefan Scholz[6], Tom Berger[1], Torben Heinsohn[3], Vitaly Belik[2], André Karch[1], Veronika K Jaeger[1]

1 Institute of Epidemiology and Social Medicine, University of Münster, Münster, Germany

2 System Modelling Group, Institute of Veterinary Epidemiology and Biostatistics, Freie Universität Berlin, Germany

3 Department of Epidemiology, Helmholtz Centre for Infection Research, Braunschweig, Germany & German Centre for Infection Research, TI BBD, Braunschweig, Germany

4 London School of Hygiene and Tropical Medicine, London, UK

5 Institute for Medical Epidemiology, Biometrics, and Informatics (IMEBI), Interdisciplinary Center for Health Sciences, Medical Faculty of the Martin Luther University Halle-Wittenberg, Halle, Germany

6 Medical Faculty of the Martin Luther University Halle-Wittenberg, Halle, Germany, until 01/2022 Immunization Unit, Infectious disease epidemiology, Robert Koch-Institute, Berlin, Germany

Corresponding author:

Veronika K Jaeger, PhD

Institute of Epidemiology and Social Medicine

University of Münster,

48149 Münster, Germany

Email: veronika.jaeger@ukmuenster.de

Keywords

Contact behaviour, heterogeneity, modelling, pandemic, SARS-CoV-2

I. Bootstrapping mean

Table AF1. The bootstrapped mean number of contacts and duration of contacts (hours) with 95%CI of all contacts, non-household contacts, and household contacts across 33 COVIMOD survey waves.

		Mean number of contacts (95%CI)			Mean duration of contacts in hours (95%CI)		
Wave	Timing	All contacts	Non-household	Household	All contacts	Non-household	Household
1	30.04.-06.05.20	2.11 (2.01, 2.22)	0.58 (0.52, 0.65)	1.53 (1.46, 1.61)	12.58 (11.84, 13.33)	1.31 (1.13, 1.52)	11.27 (10.58, 11.96)
2	14.05.-21.05.20	3.57 (3.30, 3.87)	2.13 (1.87, 2.44)	1.44 (1.36, 1.52)	15.90 (14.42, 17.48)	5.73 (4.58, 7.25)	10.17 (9.48, 10.83)
3	28.05.-04.06.20	5.60 (4.85, 6.50)	4.25 (3.50, 5.15)	1.34 (1.25, 1.44)	17.26 (15.54, 18.98)	7.51 (6.04, 9.04)	9.75 (8.95, 10.57)
4	11.06.-22.06.20	6.38 (5.67, 7.15)	5.11 (4.40, 5.88)	1.26 (1.20, 1.32)	16.62 (15.19, 17.96)	7.82 (6.63, 9.12)	8.81 (8.29, 9.33)
5	26.06.-01.07.20	4.86 (4.29, 5.49)	3.62 (3.06, 4.25)	1.24 (1.18, 1.32)	15.40 (14.32, 16.63)	6.08 (5.21, 7.02)	9.32 (8.71, 9.95)
6	09.07.-16.07.20	4.40 (3.80, 5.01)	3.14 (2.55, 3.75)	1.27 (1.20, 1.34)	15.54 (14.26, 16.88)	5.97 (4.85, 7.10)	9.58 (8.97, 10.23)
7	24.07.-29.07.20	3.48 (3.01, 4.03)	2.34 (1.86, 2.89)	1.14 (1.08, 1.22)	13.07 (12.08, 14.11)	4.25 (3.44, 5.11)	8.82 (8.22, 9.48)
8	07.08.-11.08.20	3.79 (3.06, 4.67)	2.67 (1.96, 3.57)	1.11 (1.03, 1.20)	13.95 (12.17, 16.09)	5.79 (4.23, 7.93)	8.16 (7.45, 8.89)
9	04.09.-09.09.20	3.16 (2.76, 3.63)	1.99 (1.61, 2.47)	1.17 (1.08, 1.25)	12.40 (11.24, 13.58)	3.95 (3.17, 4.85)	8.46 (7.71, 9.20)
10	30.09.-05.10.20	3.54 (2.87, 4.34)	2.51 (1.85, 3.30)	1.03 (0.93, 1.13)	12.68 (11.04, 14.45)	4.94 (3.77, 6.32)	7.74 (6.85, 8.68)
11	14.10.-21.10.20	3.60 (3.09, 4.21)	2.47 (1.98, 3.06)	1.12 (1.05, 1.20)	12.64 (11.70, 13.66)	4.46 (3.80, 5.21)	8.18 (7.60, 8.80)
12	29.10.-03.11.20	3.33 (2.88, 3.84)	2.26 (1.83, 2.76)	1.07 (1.00, 1.14)	13.08 (11.79, 14.65)	5.13 (4.00, 6.46)	7.95 (7.39, 8.58)
13	05.11.-10.11.20	2.82 (2.48, 3.22)	1.77 (1.45, 2.14)	1.05 (0.98, 1.12)	12.31 (11.28, 13.44)	4.23 (3.43, 5.09)	8.08 (7.52, 8.69)
14	25.11.-30.11.20	2.77 (2.44, 3.16)	1.71 (1.39, 2.08)	1.06 (1.00, 1.12)	10.70 (9.71, 11.81)	3.59 (2.84, 4.51)	7.11 (6.61, 7.64)
15	09.12.-15.12.20	2.69 (2.34, 3.11)	1.69 (1.36, 2.09)	1.00 (0.95, 1.06)	9.78 (8.86, 10.81)	3.13 (2.46, 3.91)	6.65 (6.16, 7.21)
16	23.12.-30.12.20	2.35 (2.03, 2.73)	1.31 (0.99, 1.69)	1.04 (0.98, 1.11)	10.52 (9.67, 11.49)	2.71 (2.08, 3.52)	7.82 (7.28, 8.38)
17	28.01.-02.02.21	2.56 (2.03, 3.20)	1.54 (1.01, 2.19)	1.02 (0.94, 1.09)	10.27 (8.90, 11.90)	3.08 (2.02, 4.48)	7.19 (6.49, 7.86)
18	24.02.-03.03.21	2.49 (2.15, 2.86)	1.43 (1.11, 1.80)	1.05 (0.99, 1.11)	11.28 (9.94, 12.68)	3.96 (2.82, 5.20)	7.32 (6.79, 7.88)
19	17.03.-26.03.21	2.85 (2.36, 3.43)	1.82 (1.33, 2.40)	1.03 (0.96, 1.09)	11.25 (9.88, 12.73)	4.01 (2.84, 5.41)	7.24 (6.68, 7.79)
20	07.04.-15.04.21	2.34 (2.03, 2.70)	1.34 (1.04, 1.69)	1.00 (0.94, 1.06)	9.73 (8.95, 10.63)	2.54 (1.96, 3.24)	7.19 (6.65, 7.71)
21	12.05.-24.05.21	3.05 (2.73, 3.39)	2.04 (1.74, 2.38)	1.00 (0.95, 1.05)	11.50 (10.58, 12.46)	4.67 (3.85, 5.55)	6.83 (6.38, 7.19)
22	26.05.-03.06.21	2.78 (2.53, 3.08)	1.75 (1.50, 2.04)	1.03 (0.99, 1.08)	11.17 (10.42, 12.01)	3.93 (3.27, 4.64)	7.24 (6.85, 7.64)
23	09.06.-22.06.21	3.00 (2.70, 3.35)	1.99 (1.70, 2.34)	1.01 (0.96, 1.06)	12.01 (11.03, 13.00)	4.93 (4.09, 5.80)	7.09 (6.70, 7.49)
24	07.07.-19.07.21	3.22 (2.80, 3.66)	2.22 (1.81, 2.65)	1.00 (0.95, 1.04)	11.03 (10.27, 11.82)	3.99 (3.37, 4.61)	7.04 (6.66, 7.45)
25	04.08.-13.08.21	3.00 (2.69, 3.32)	2.05 (1.75, 2.37)	0.95 (0.91, 0.99)	11.10 (10.28, 12.05)	4.18 (3.51, 5.00)	6.92 (6.52, 7.32)
26	01.09.-14.09.21	3.24 (2.92, 3.59)	2.28 (1.98, 2.63)	0.96 (0.92, 1.00)	11.93 (11.04, 12.94)	5.20 (4.39, 6.07)	6.74 (6.40, 7.13)
27	22.09.-06.10.21	3.84 (3.48, 4.26)	2.85 (2.49, 3.26)	0.99 (0.95, 1.04)	13.62 (12.58, 14.77)	6.78 (5.84, 7.81)	6.84 (6.47, 7.25)
28	08.10.-20.10.21	3.54 (3.18, 3.94)	2.57 (2.20, 2.96)	0.97 (0.93, 1.02)	12.78 (11.76, 13.82)	6.14 (5.19, 7.10)	6.64 (6.28, 7.00)
29	22.10.-02.11.21	3.36 (3.05, 3.72)	2.42 (2.10, 2.79)	0.95 (0.90, 0.99)	12.21 (11.28, 13.22)	5.65 (4.83, 6.54)	6.56 (6.19, 6.92)
30	03.11.-09.11.21	3.29 (2.96, 3.67)	2.31 (2.00, 2.69)	0.98 (0.94, 1.02)	12.16 (11.15, 13.24)	5.48 (4.59, 6.49)	6.68 (6.33, 7.03)
31	17.11.-23.11.21	3.17 (2.83, 3.53)	2.22 (1.89, 2.57)	0.95 (0.91, 0.99)	11.98 (10.94, 13.07)	5.55 (4.60, 6.53)	6.43 (6.09, 6.78)
32	08-12.-17.12.21	3.21 (2.93, 3.51)	2.24 (1.96, 2.54)	0.98 (0.93, 1.02)	11.56 (10.64, 12.45)	4.97 (4.16, 5.76)	6.58 (6.20, 6.97)
33	24-12.-31.12.21	2.49 (2.19, 2.84)	1.53 (1.24, 1.88)	0.96 (0.92, 1.00)	10.03 (9.52, 10.59)	3.03 (2.64, 3.46)	6.99 (6.64, 7.34)
Overall		3.30 (3.23, 3.38)	2.24 (2.17, 2.32)	1.06 (1.05, 1.07)	12.28 (12.08, 12.47)	4.76 (4.59, 4.93)	7.52 (7.43, 7.60)

II. Generalized additive models (GAMs)

1. All participants

Table AF2. The estimated coefficients of factors influencing the daily number of contacts and duration of contacts per person in Germany in all settings and specified settings, including work, school, home or leisure, and other settings (GAM models applied to all participants).

	All	Work	School	Home/Leisure	Other
**Contact Number Ratio (CNR)**					
**Demographic characteristic. Age ref. 20-34**					
Age: 0-19	1.36 (1.30, 1.42)	0.22 (0.18, 0.26)	9.58 (7.91, 11.61)	1.37 (1.33, 1.41)	1.33 (1.20, 1.48)
Age: 35-44	1.07 (1.02, 1.12)	0.89 (0.75, 1.05)	0.21 (0.16, 0.27)	1.05 (1.01, 1.09)	1.08 (0.97, 1.21)
Age: 45-54	1.16 (1.11, 1.21)	1.17 (0.99, 1.37)	0.22 (0.17, 0.28)	1.12 (1.09, 1.17)	1.28 (1.15, 1.42)
Age: 55-64	1.15 (1.10, 1.20)	0.87 (0.74, 1.02)	0.15 (0.11, 0.19)	1.29 (1.25, 1.34)	1.40 (1.26, 1.56)
Age: 65+	0.96 (0.92, 1.00)	0.31 (0.26, 0.36)	0.08 (0.06, 0.11)	1.43 (1.39, 1.48)	1.42 (1.29, 1.58)
Female	0.94 (0.92, 0.96)	0.74 (0.68, 0.81)	0.76 (0.67, 0.87)	1.04 (1.02, 1.06)	0.77 (0.73, 0.82)
No. household members	1.18 (1.17, 1.19)	1.00 (0.96, 1.04)	1.19 (1.13, 1.26)	1.39 (1.38, 1.40)	1.01 (0.98, 1.03)
**Containment measures**					
School closure	0.79 (0.77, 0.81)	0.58 (0.52, 0.63)	0.47 (0.41, 0.54)	1.07 (1.05, 1.09)	0.77 (0.72, 0.82)
Social gathering limitations	0.90 (0.87, 0.94)	1.02 (0.88, 1.18)	0.89 (0.72, 1.10)	0.99 (0.96, 1.02)	0.84 (0.77, 0.92)
**Risk perception (Likely to)**					
Catch COVID	1.11 (1.05, 1.17)	1.47 (1.18, 1.82)	0.66 (0.47, 0.93)	0.90 (0.86, 0.94)	1.22 (1.06, 1.40)
Get severe illness	0.99 (0.95, 1.03)	1.11 (0.97, 1.28)	0.55 (0.43, 0.69)	0.96 (0.94, 0.99)	1.10 (1.01, 1.21)
Spread COVID	1.05 (1.02, 1.08)	1.13 (1.01, 1.26)	0.94 (0.79, 1.11)	1.08 (1.06, 1.11)	1.04 (0.97, 1.12)
**Personal health**					
COVID symptoms	1.30 (1.26, 1.34)	1.43 (1.28, 1.59)	1.80 (1.54, 2.11)	1.13 (1.11, 1.16)	1.77 (1.65, 1.90)
Risk severe disease	0.92 (0.90, 0.95)	0.82 (0.73, 0.92)	0.67 (0.56, 0.81)	1.02 (1.00, 1.04)	1.07 (0.99, 1.15)
**Contact Duration (hours) Ratio (CDR)**					
**Demographic characteristic. Age ref. 20-34**					
Age: 0-19	1.43 (1.36, 1.50)	0.22 (0.18, 0.27)	13.19 (10.35, 16.82)	1.23 (1.17, 1.29)	1.59 (1.40, 1.81)
Age: 35-44	1.11 (1.06, 1.17)	0.93 (0.76, 1.13)	0.16 (0.12, 0.21)	1.09 (1.04, 1.15)	1.11 (0.97, 1.27)
Age: 45-54	1.20 (1.14, 1.26)	1.14 (0.94, 1.38)	0.21 (0.16, 0.27)	1.22 (1.17, 1.29)	1.38 (1.21, 1.57)
Age: 55-64	1.24 (1.18, 1.30)	0.84 (0.69, 1.01)	0.11 (0.08, 0.14)	1.49 (1.42, 1.56)	1.49 (1.31, 1.70)
Age: 65+	1.12 (1.07, 1.17)	0.25 (0.21, 0.30)	0.08 (0.06, 0.11)	1.61 (1.54, 1.69)	1.59 (1.40, 1.79)
Female	1.03 (1.00, 1.05)	0.74 (0.67, 0.83)	0.75 (0.64, 0.88)	1.08 (1.05, 1.11)	0.90 (0.84, 0.97)
No. household members	1.47 (1.45, 1.48)	1.04 (0.99, 1.09)	1.33 (1.24, 1.42)	1.82 (1.80, 1.85)	1.22 (1.18, 1.27)
**Containment measures**					
School closure	0.90 (0.87, 0.92)	0.57 (0.52, 0.64)	0.41 (0.35, 0.48)	1.15 (1.12, 1.18)	0.95 (0.89, 1.02)
Social gathering limitations	0.94 (0.90, 0.98)	0.95 (0.81, 1.13)	0.93 (0.73, 1.19)	1.01 (0.97, 1.05)	0.91 (0.82, 1.01)
**Risk perception (Likely to)**					
Catch COVID	0.99 (0.93, 1.06)	1.38 (1.07, 1.78)	0.50 (0.33, 0.75)	0.90 (0.84, 0.96)	1.04 (0.88, 1.23)
Get severe illness	1.04 (0.99, 1.08)	1.06 (0.90, 1.24)	0.51 (0.39, 0.66)	0.99 (0.95, 1.03)	1.15 (1.04, 1.28)
Spread COVID	1.10 (1.06, 1.14)	1.19 (1.04, 1.36)	1.08 (0.89, 1.31)	1.10 (1.06, 1.14)	1.14 (1.05, 1.25)
**Personal health**					
COVID symptoms	1.07 (1.03, 1.10)	1.26 (1.11, 1.44)	1.82 (1.51, 2.19)	1.01 (0.97, 1.04)	1.47 (1.35, 1.60)
Risk severe disease	0.93 (0.90, 0.97)	0.87 (0.76, 0.99)	0.39 (0.32, 0.48)	0.99 (0.96, 1.03)	1.08 (0.99, 1.18)

2. Participants aged 18 and under

Figure AF1. The Contact Number Ratio (CNR) or Contact Duration Ratio (CDR) among individuals aged 18 or under at a specified setting, including all settings, school, home or leisure, and other settings. Dots are the point estimates from GAM models; horizontal lines are 95% confidence intervals.
These estimates were derived from multivariable analysis. The reference groups for comparison are 0-5 for age groups; male for sex; less than 30% closing for school closure; no measures in place for social gathering limitations (e.g. workplace restriction or requiring a stay-at-home); “strongly agree” compared to a less strongly opinionated group (which includes "strongly disagree", "tend to disagree", "neutral", and "tend to agree") for risk perceptions; no COVID-19 symptoms and no risk for severe disease if contracted COVID-19 for personal health. For the grouping of the contact settings into home/leisure and other, please look at Additional file 2 Table AF14.

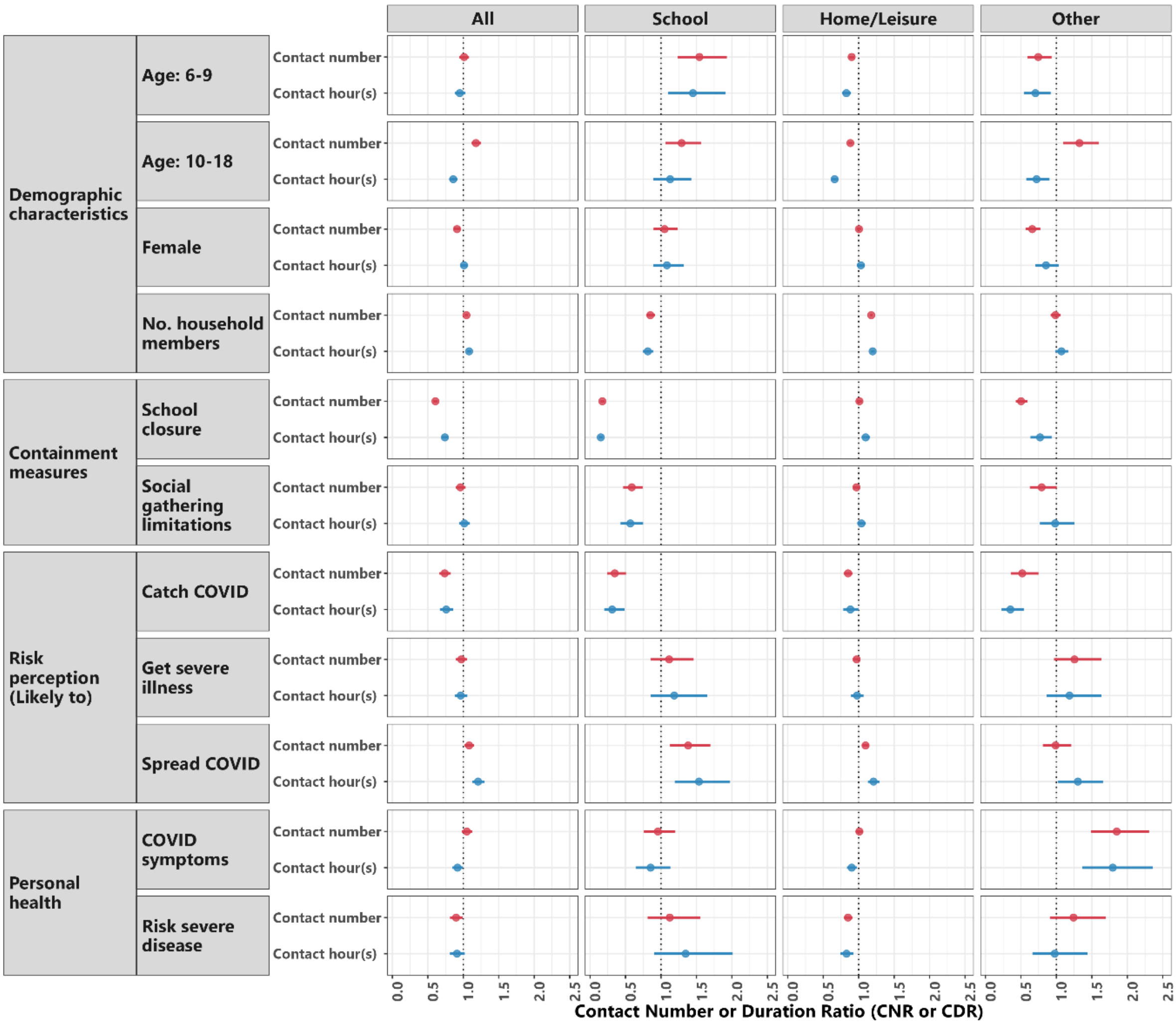

Table AF3. The estimated coefficients of factors influencing the daily number of contacts and duration of contacts per person in Germany in all settings and specified settings, including school, home or leisure, and other settings (GAM models applied to participants under 19 years old).

	All	School	Home/Leisure	Other
**Contact Number Ratio (CNR)**				
**Demographic characteristic. Age ref. 0-5**				
Age: 6-9	1.01 (0.94, 1.08)	1.54 (1.23, 1.93)	0.90 (0.86, 0.94)	0.74 (0.59, 0.93)
Age: 10-18	1.18 (1.11, 1.25)	1.29 (1.06, 1.56)	0.88 (0.85, 0.92)	1.32 (1.10, 1.60)
Female	0.91 (0.87, 0.96)	1.05 (0.89, 1.23)	1.00 (0.97, 1.04)	0.66 (0.56, 0.77)
No. household members	1.04 (1.02, 1.07)	0.85 (0.79, 0.92)	1.18 (1.16, 1.19)	0.99 (0.92, 1.06)
**Containment measures**				
School closure	0.61 (0.58, 0.64)	0.17 (0.15, 0.20)	1.01 (0.98, 1.04)	0.50 (0.43, 0.59)
Social gathering limitations	0.96 (0.89, 1.03)	0.59 (0.47, 0.74)	0.97 (0.92, 1.02)	0.79 (0.63, 1.00)
**Risk perception (Likely to)**				
Catch COVID	0.74 (0.66, 0.82)	0.35 (0.24, 0.51)	0.85 (0.79, 0.92)	0.52 (0.36, 0.75)
Get severe illness	0.97 (0.90, 1.05)	1.12 (0.85, 1.46)	0.97 (0.92, 1.03)	1.26 (0.97, 1.63)
Spread COVID	1.08 (1.02, 1.15)	1.38 (1.13, 1.70)	1.10 (1.05, 1.14)	0.99 (0.81, 1.21)
**Personal health**				
COVID symptoms	1.05 (0.98, 1.13)	0.96 (0.76, 1.20)	1.01 (0.96, 1.06)	1.85 (1.49, 2.31)
Risk severe disease	0.90 (0.81, 0.99)	1.12 (0.81, 1.56)	0.85 (0.79, 0.91)	1.24 (0.91, 1.70)
**Contact Duration (hours) Ratio (CDR)**				
**Demographic characteristic. Age ref. 0-5**				
Age: 6-9	0.95 (0.88, 1.03)	1.45 (1.10, 1.91)	0.83 (0.77, 0.89)	0.70 (0.54, 0.92)
Age: 10-18	0.86 (0.80, 0.92)	1.13 (0.89, 1.43)	0.66 (0.62, 0.70)	0.72 (0.57, 0.90)
Female	1.01 (0.96, 1.07)	1.08 (0.89, 1.32)	1.03 (0.98, 1.09)	0.85 (0.71, 1.03)
No. household members	1.08 (1.05, 1.11)	0.81 (0.74, 0.89)	1.20 (1.17, 1.23)	1.07 (0.98, 1.17)
**Containment measures**				
School closure	0.74 (0.70, 0.78)	0.15 (0.12, 0.18)	1.10 (1.04, 1.16)	0.77 (0.63, 0.93)
Social gathering limitations	1.01 (0.94, 1.09)	0.57 (0.43, 0.75)	1.04 (0.98, 1.10)	0.98 (0.77, 1.26)
**Risk perception (Likely to)**				
Catch COVID	0.76 (0.67, 0.86)	0.31 (0.20, 0.49)	0.88 (0.78, 1.00)	0.35 (0.23, 0.54)
Get severe illness	0.96 (0.88, 1.06)	1.19 (0.85, 1.65)	0.98 (0.89, 1.07)	1.18 (0.86, 1.63)
Spread COVID	1.21 (1.13, 1.30)	1.54 (1.19, 1.97)	1.21 (1.13, 1.29)	1.30 (1.02, 1.66)
**Personal health**				
COVID symptoms	0.92 (0.85, 1.00)	0.85 (0.64, 1.13)	0.90 (0.83, 0.97)	1.80 (1.37, 2.36)
Risk severe disease	0.91 (0.81, 1.02)	1.35 (0.90, 2.01)	0.83 (0.74, 0.93)	0.98 (0.66, 1.44)

3. Participants aged over 18

Figure AF2. The Contact Number Ratio (CNR) or Contact Duration Ratio (CDR) among individuals aged over 18 at a specified setting, including all settings, work, home or leisure, and other settings in Germany. Dots are the point estimates from GAM models; horizontal lines are 95% confidence intervals.

These estimates were derived from multivariable analysis. The reference groups for comparison are 19-34 for age groups; male for sex; less than 30% closing for school closure; no measures in place for social gathering limitations (e.g. workplace restriction or requiring a stay-at-home); “strongly agree” compared to a less strongly opinionated group (which includes "strongly disagree", "tend to disagree", "neutral", and "tend to agree") for risk perceptions; no COVID-19 symptoms and no risk for severe disease if contracted COVID-19 for personal health. For the grouping of the contact settings into home/leisure and other, please look at Additional file 2 Table AF14.

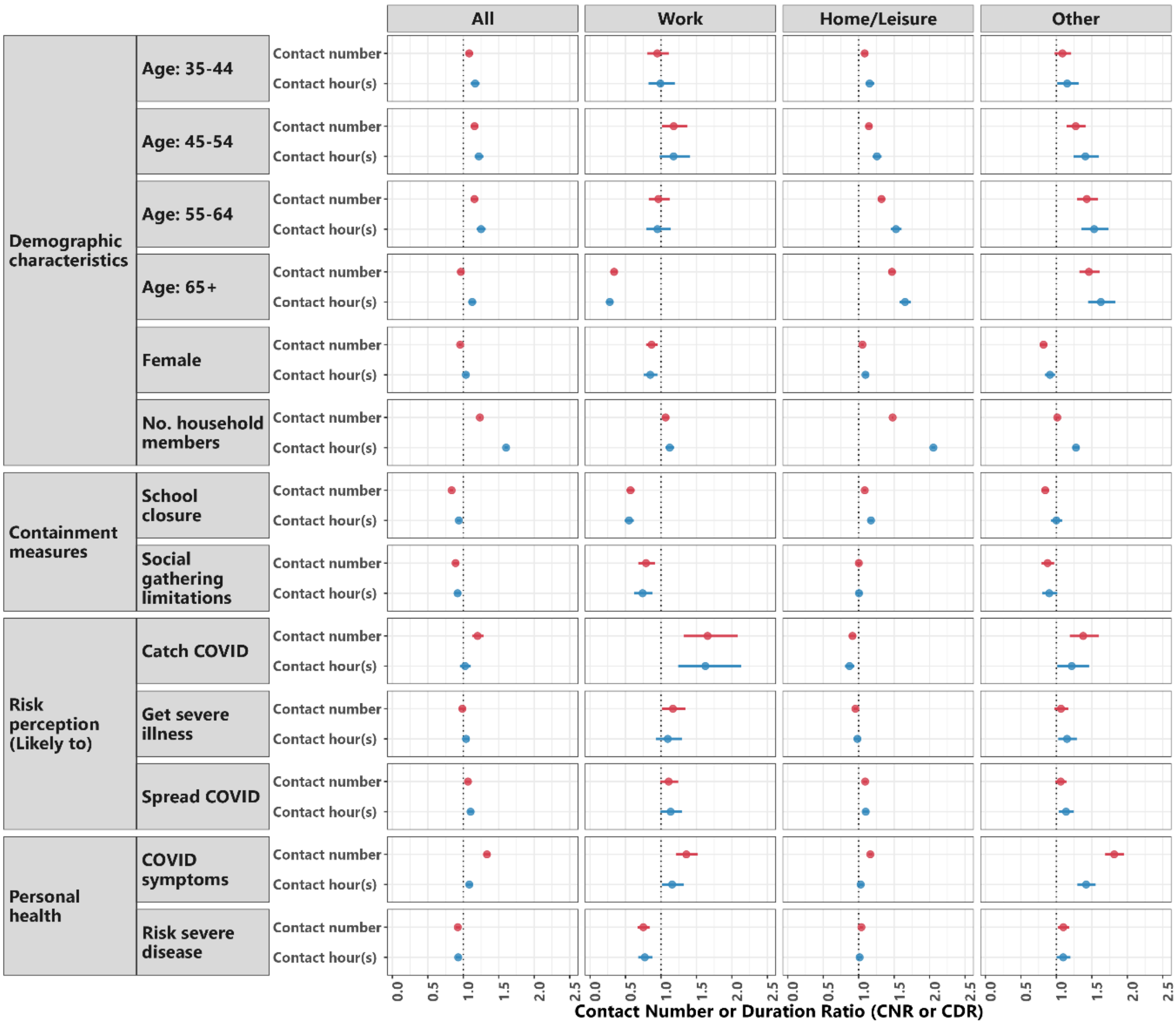

Table AF4. The estimated coefficients of factors influencing the daily number of contacts and duration of contacts per person in Germany in all settings and specified settings, including work, home or leisure, and other settings (GAM models applied to participants over 18 years old).

	All	Work	Home/Leisure	Other
**Contact Number Ratio (CNR)**				
**Demographic characteristic. Age ref. 19-34**				
Age: 35-44	1.08 (1.03, 1.14)	0.95 (0.81, 1.11)	1.08 (1.04, 1.13)	1.09 (0.98, 1.21)
Age: 45-54	1.16 (1.11, 1.21)	1.18 (1.01, 1.37)	1.14 (1.10, 1.19)	1.27 (1.15, 1.41)
Age: 55-64	1.16 (1.11, 1.21)	0.96 (0.83, 1.12)	1.32 (1.27, 1.37)	1.43 (1.29, 1.58)
Age: 65+	0.97 (0.92, 1.01)	0.34 (0.29, 0.39)	1.47 (1.42, 1.52)	1.46 (1.32, 1.61)
Female	0.96 (0.93, 0.98)	0.87 (0.79, 0.95)	1.05 (1.03, 1.08)	0.82 (0.77, 0.87)
No. household members	1.23 (1.22, 1.25)	1.06 (1.02, 1.11)	1.48 (1.47, 1.49)	1.01 (0.98, 1.04)
**Containment measures**				
School closure	0.84 (0.81, 0.86)	0.57 (0.52, 0.63)	1.09 (1.06, 1.11)	0.84 (0.79, 0.90)
Social gathering limitations	0.89 (0.85, 0.93)	0.79 (0.68, 0.92)	1.00 (0.97, 1.04)	0.88 (0.79, 0.97)
**Risk perception (Likely to)**				
Catch COVID	1.20 (1.12, 1.28)	1.66 (1.32, 2.08)	0.91 (0.87, 0.96)	1.38 (1.19, 1.60)
Get severe illness	0.99 (0.95, 1.03)	1.17 (1.01, 1.35)	0.95 (0.92, 0.98)	1.06 (0.97, 1.17)
Spread COVID	1.07 (1.03, 1.10)	1.11 (0.99, 1.24)	1.09 (1.06, 1.12)	1.06 (0.98, 1.15)
**Personal health**				
COVID symptoms	1.33 (1.29, 1.38)	1.36 (1.21, 1.52)	1.16 (1.13, 1.19)	1.82 (1.69, 1.95)
Risk severe disease	0.92 (0.89, 0.95)	0.75 (0.67, 0.84)	1.04 (1.01, 1.06)	1.10 (1.02, 1.18)
**Contact Duration (hours) Ratio (CDR)**				
**Demographic characteristic. Age ref. 19-34**				
Age: 35-44	1.17 (1.11, 1.23)	0.99 (0.82, 1.19)	1.15 (1.09, 1.22)	1.15 (1.01, 1.31)
Age: 45-54	1.22 (1.16, 1.28)	1.18 (0.98, 1.41)	1.26 (1.20, 1.32)	1.41 (1.24, 1.60)
Age: 55-64	1.25 (1.19, 1.32)	0.95 (0.79, 1.14)	1.53 (1.45, 1.60)	1.53 (1.35, 1.74)
Age: 65+	1.13 (1.07, 1.18)	0.28 (0.23, 0.33)	1.65 (1.58, 1.73)	1.63 (1.45, 1.83)
Female	1.04 (1.00, 1.07)	0.85 (0.76, 0.95)	1.10 (1.06, 1.13)	0.91 (0.84, 0.98)
No. household members	1.60 (1.58, 1.63)	1.12 (1.07, 1.18)	2.05 (2.02, 2.08)	1.28 (1.23, 1.32)
**Containment measures**				
School closure	0.94 (0.91, 0.97)	0.55 (0.49, 0.62)	1.17 (1.14, 1.21)	1.00 (0.92, 1.08)
Social gathering limitations	0.92 (0.88, 0.97)	0.74 (0.62, 0.88)	1.00 (0.96, 1.05)	0.90 (0.80, 1.01)
**Risk perception (Likely to)**				
Catch COVID	1.03 (0.95, 1.11)	1.63 (1.24, 2.13)	0.87 (0.81, 0.94)	1.22 (1.01, 1.46)
Get severe illness	1.04 (0.99, 1.09)	1.09 (0.93, 1.29)	0.98 (0.94, 1.03)	1.15 (1.03, 1.29)
Spread COVID	1.10 (1.06, 1.15)	1.14 (0.99, 1.30)	1.10 (1.06, 1.14)	1.14 (1.04, 1.25)
**Personal health**				
COVID symptoms	1.08 (1.05, 1.13)	1.16 (1.01, 1.32)	1.03 (0.99, 1.07)	1.42 (1.30, 1.55)
Risk severe disease	0.93 (0.90, 0.96)	0.77 (0.68, 0.88)	1.01 (0.98, 1.05)	1.10 (1.00, 1.20)

III. Non-household contact characteristics

Table AF5. Percentage and bootstrap mean number of non-household contacts (with 95%CI) **at work, school** (include contacts at childcare, schools, and universities), **home or leisure places** (including contacts at home; at somebody else's place; at a place of worship; at a shop for non-essential items; at a place of entertainment, e.g. bar, restaurant, cinema; at a place of sport, e.g. gym, sports club; at an outside place, e.g. a park; at a beauty place, e.g. hairdresser, nail salon), and **other settings** (including transport; healthcare setting, e.g. hospital, GP, dentist; shop for essential goods: somewhere else; and at an unspecified location).

			Percentange (%)				Mean number of contacts (95%CI)			
Wave	Timing	N	Work	School	Leisure	Other	Work	School	Leisure	Other
1	30.04.-06.05.20	993	22.66	0.60	55.99	20.75	0.15 (0.11, 0.19)	0.00 (0.00, 0.01)	0.35 (0.31, 0.41)	0.13 (0.10, 0.15)
2	14.05.-21.05.20	3146	39.96	1.88	32.49	25.68	0.88 (0.67, 1.15)	0.05 (0.03, 0.07)	0.76 (0.67, 0.85)	0.56 (0.46, 0.68)
3	28.05.-04.06.20	4663	41.50	2.55	18.46	37.49	1.79 (1.23, 2.43)	0.17 (0.09, 0.28)	0.78 (0.67, 0.90)	1.68 (1.22, 2.20)
4	11.06.-22.06.20	9897	40.57	6.53	17.03	35.88	2.13 (1.61, 2.67)	0.39 (0.23, 0.60)	0.88 (0.78, 0.99)	1.89 (1.53, 2.32)
5	26.06.-01.07.20	6004	35.51	5.06	22.25	37.18	1.30 (0.97, 1.69)	0.24 (0.15, 0.37)	0.83 (0.73, 0.93)	1.42 (1.07, 1.83)
6	09.07.-16.07.20	4856	42.50	3.32	21.89	32.29	1.36 (0.95, 1.78)	0.12 (0.07, 0.18)	0.71 (0.62, 0.79)	1.12 (0.79, 1.48)
7	24.07.-29.07.20	3072	36.65	2.99	25.36	34.99	0.86 (0.54, 1.20)	0.08 (0.03, 0.15)	0.66 (0.58, 0.76)	0.92 (0.67, 1.23)
8	07.08.-11.08.20	2978	36.40	2.65	21.93	39.02	1.04 (0.61, 1.61)	0.08 (0.02, 0.17)	0.63 (0.52, 0.74)	1.12 (0.75, 1.56)
9	04.09.-09.09.20	1851	30.47	5.89	30.09	33.55	0.66 (0.39, 1.00)	0.13 (0.08, 0.20)	0.62 (0.50, 0.76)	0.71 (0.53, 0.95)
10	30.09.-05.10.20	1921	33.16	7.50	19.94	39.41	0.89 (0.46, 1.41)	0.27 (0.15, 0.42)	0.50 (0.41, 0.61)	0.98 (0.62, 1.40)
11	14.10.-21.10.20	3643	30.61	9.44	22.89	37.06	0.75 (0.55, 0.98)	0.27 (0.12, 0.48)	0.54 (0.48, 0.61)	1.04 (0.66, 1.48)
12	29.10.-03.11.20	3493	43.20	4.87	23.73	28.20	0.98 (0.66, 1.35)	0.15 (0.08, 0.24)	0.56 (0.46, 0.67)	0.69 (0.47, 0.98)
13	05.11.-10.11.20	2823	35.46	11.02	21.71	31.81	0.68 (0.47, 0.93)	0.23 (0.13, 0.35)	0.40 (0.34, 0.46)	0.60 (0.41, 0.82)
14	25.11.-30.11.20	2596	35.63	12.98	21.38	30.01	0.64 (0.45, 0.86)	0.28 (0.16, 0.43)	0.38 (0.32, 0.43)	0.54 (0.32, 0.83)
15	09.12.-15.12.20	2692	34.70	15.75	18.05	31.50	0.59 (0.38, 0.86)	0.33 (0.21, 0.50)	0.32 (0.27, 0.38)	0.55 (0.35, 0.80)
16	23.12.-30.12.20	2062	35.69	1.16	34.24	28.90	0.47 (0.25, 0.78)	0.02 (0.00, 0.07)	0.47 (0.40, 0.54)	0.39 (0.24, 0.62)
17	28.01.-02.02.21	1534	53.91	3.85	18.19	24.05	0.85 (0.39, 1.41)	0.08 (0.02, 0.15)	0.28 (0.23, 0.33)	0.39 (0.21, 0.66)
18	24.02.-03.03.21	2161	40.49	12.08	24.29	23.14	0.58 (0.33, 0.91)	0.23 (0.12, 0.35)	0.35 (0.29, 0.42)	0.34 (0.24, 0.45)
19	17.03.-26.03.21	2839	41.88	13.28	16.87	27.97	0.78 (0.41, 1.26)	0.30 (0.19, 0.43)	0.33 (0.27, 0.41)	0.53 (0.32, 0.79)
20	07.04.-15.04.21	2079	40.21	3.56	27.13	29.10	0.55 (0.32, 0.83)	0.07 (0.02, 0.14)	0.37 (0.31, 0.44)	0.41 (0.26, 0.62)
21	12.05.-24.05.21	5159	36.44	16.50	21.83	25.24	0.77 (0.59, 0.98)	0.36 (0.25, 0.48)	0.47 (0.41, 0.52)	0.52 (0.40, 0.67)
22	26.05.-03.06.21	4458	29.05	13.89	30.96	26.11	0.54 (0.39, 0.71)	0.29 (0.19, 0.39)	0.57 (0.50, 0.64)	0.47 (0.35, 0.62)
23	09.06.-22.06.21	5286	29.44	18.86	29.40	22.30	0.61 (0.45, 0.83)	0.45 (0.30, 0.62)	0.61 (0.51, 0.74)	0.45 (0.35, 0.60)
24	07.07.-19.07.21	5715	34.66	18.02	23.10	24.22	0.79 (0.55, 1.06)	0.48 (0.30, 0.70)	0.53 (0.47, 0.59)	0.57 (0.42, 0.75)
25	04.08.-13.08.21	5432	36.03	7.40	29.77	26.80	0.80 (0.61, 1.01)	0.20 (0.11, 0.31)	0.64 (0.56, 0.73)	0.58 (0.44, 0.74)
26	01.09.-14.09.21	6203	31.16	12.85	30.37	25.62	0.78 (0.60, 0.99)	0.39 (0.24, 0.56)	0.76 (0.64, 0.91)	0.60 (0.47, 0.75)
27	22.09.-06.10.21	7423	31.42	18.32	23.05	27.21	0.92 (0.74, 1.15)	0.65 (0.49, 0.82)	0.67 (0.57, 0.79)	0.73 (0.56, 0.93)
28	08.10.-20.10.21	6752	32.24	18.78	23.09	25.89	0.86 (0.67, 1.09)	0.60 (0.44, 0.78)	0.60 (0.53, 0.67)	0.67 (0.50, 0.89)
29	22.10.-02.11.21	6328	28.32	17.83	28.59	25.27	0.73 (0.58, 0.90)	0.49 (0.32, 0.72)	0.74 (0.65, 0.84)	0.62 (0.49, 0.77)
30	03.11.-09.11.21	5963	32.90	18.41	26.46	22.22	0.83 (0.62, 1.07)	0.46 (0.33, 0.61)	0.64 (0.55, 0.74)	0.52 (0.41, 0.65)
31	17.11.-23.11.21	5795	35.32	19.83	22.92	21.93	0.85 (0.63, 1.12)	0.49 (0.36, 0.62)	0.55 (0.45, 0.67)	0.50 (0.38, 0.63)
32	08-12.-17.12.21	5835	32.99	16.32	22.38	28.31	0.76 (0.57, 0.95)	0.45 (0.34, 0.58)	0.51 (0.45, 0.58)	0.62 (0.49, 0.77)
33	24-12.-31.12.21	4149	31.48	2.75	39.53	26.25	0.53 (0.33, 0.77)	0.05 (0.02, 0.10)	0.66 (0.59, 0.72)	0.42 (0.28, 0.59)
Overall		139801	35.14	11.35	24.73	28.78	0.82 (0.77, 0.87)	0.31 (0.28, 0.33)	0.58 (0.56, 0.60)	0.67 (0.64, 0.71)

Table AF6. The percentage and bootstrap mean number of non-household contacts (with 95%CI) stratified by contact types (**physical**, (e.g., handshaking, hugging, kissing) and **non-physical**).

			Percentage (%)		Mean number of contacts (95% CI)	
**Wave**	**Timing**	**N**	**Physical (%)**	**Non-physical (%)**	**Physical**	**Non-physical**
1	30.04.-06.05.20	895	18.21	81.79	0.11 (0.08, 0.13)	0.46 (0.40, 0.53)
2	14.05.-21.05.20	2883	15.92	84.08	0.34 (0.23, 0.52)	1.70 (1.49, 1.96)
3	28.05.-04.06.20	4393	9.90	90.10	0.45 (0.32, 0.60)	3.70 (3.00, 4.51)
4	11.06.-22.06.20	9426	13.78	86.22	0.70 (0.52, 0.89)	4.36 (3.70, 5.05)
5	26.06.-01.07.20	5647	10.59	89.41	0.40 (0.32, 0.48)	3.17 (2.61, 3.79)
6	09.07.-16.07.20	4552	13.88	86.12	0.45 (0.30, 0.65)	2.65 (2.13, 3.22)
7	24.07.-29.07.20	2840	15.81	84.19	0.39 (0.28, 0.53)	1.94 (1.50, 2.41)
8	07.08.-11.08.20	2675	18.43	81.57	0.53 (0.24, 0.99)	2.05 (1.56, 2.65)
9	04.09.-09.09.20	1701	15.81	84.19	0.31 (0.22, 0.42)	1.65 (1.30, 2.10)
10	30.09.-05.10.20	1823	13.49	86.51	0.36 (0.25, 0.48)	2.14 (1.54, 2.88)
11	14.10.-21.10.20	3431	20.49	79.51	0.51 (0.35, 0.75)	1.94 (1.54, 2.41)
12	29.10.-03.11.20	3306	13.40	86.60	0.33 (0.23, 0.46)	1.91 (1.49, 2.37)
13	05.11.-10.11.20	2579	14.04	85.96	0.26 (0.19, 0.33)	1.49 (1.19, 1.86)
14	25.11.-30.11.20	2384	16.11	83.89	0.29 (0.20, 0.40)	1.39 (1.09, 1.74)
15	09.12.-15.12.20	2444	12.81	87.19	0.23 (0.13, 0.38)	1.40 (1.11, 1.76)
16	23.12.-30.12.20	1942	17.61	82.39	0.24 (0.17, 0.31)	1.04 (0.76, 1.40)
17	28.01.-02.02.21	1470	15.31	84.69	0.23 (0.13, 0.38)	1.30 (0.79, 1.93)
18	24.02.-03.03.21	2003	18.12	81.88	0.28 (0.19, 0.38)	1.12 (0.83, 1.47)
19	17.03.-26.03.21	2603	14.21	85.79	0.27 (0.17, 0.38)	1.52 (1.08, 2.08)
20	07.04.-15.04.21	1945	17.94	82.06	0.26 (0.16, 0.41)	1.04 (0.79, 1.35)
21	12.05.-24.05.21	4668	17.40	82.60	0.36 (0.28, 0.46)	1.56 (1.31, 1.87)
22	26.05.-03.06.21	3901	21.87	78.13	0.38 (0.30, 0.46)	1.26 (1.05, 1.50)
23	09.06.-22.06.21	4703	18.22	81.78	0.37 (0.29, 0.46)	1.53 (1.28, 1.83)
24	07.07.-19.07.21	5200	16.58	83.42	0.37 (0.30, 0.45)	1.79 (1.40, 2.21)
25	04.08.-13.08.21	4773	18.33	81.67	0.36 (0.30, 0.43)	1.58 (1.31, 1.88)
26	01.09.-14.09.21	5395	22.74	77.26	0.53 (0.42, 0.64)	1.67 (1.41, 1.96)
27	22.09.-06.10.21	6777	14.86	85.14	0.45 (0.36, 0.54)	2.24 (1.93, 2.61)
28	08.10.-20.10.21	6016	21.88	78.12	0.56 (0.41, 0.74)	1.87 (1.59, 2.17)
29	22.10.-02.11.21	5666	24.11	75.89	0.58 (0.47, 0.68)	1.73 (1.47, 2.04)
30	03.11.-09.11.21	5433	19.36	80.64	0.43 (0.35, 0.51)	1.81 (1.51, 2.15)
31	17.11.-23.11.21	5188	20.45	79.55	0.45 (0.34, 0.56)	1.68 (1.41, 1.99)
32	08-12.-17.12.21	5408	18.03	81.97	0.42 (0.34, 0.53)	1.75 (1.50, 2.01)
33	24-12.-31.12.21	3781	29.28	70.72	0.47 (0.36, 0.59)	1.04 (0.81, 1.33)
Overall		127851	17.42	82.58	0.40 (0.38, 0.42)	1.77 (1.71, 1.84)

Table AF7. The percentage and bootstrap mean number of non-household contacts and duration (hours) of the contacts (with 95%CI) stratified by a contact's location (inside or outside).

			Percentage (%)		Mean number of contacts (95% CI)		Mean duration of contacts in hours (95% CI)	
**Wave**	**Timing**	**N**	**Inside**	**Outside**	**Inside**	**Outside**	**Inside**	**Outside**
1	30.04.-06.05.20	953	72.93	27.07	0.44 (0.39, 0.50)	0.16 (0.14, 0.20)	1.15 (0.97, 1.35)	0.46 (0.36, 0.58)
2	14.05.-21.05.20	3085	78.87	21.13	1.73 (1.47, 2.02)	0.49 (0.41, 0.58)	5.15 (4.01, 6.66)	1.30 (1.02, 1.62)
3	28.05.-04.06.20	2399	76.37	23.63	1.58 (1.34, 1.85)	0.55 (0.44, 0.70)	4.40 (3.48, 5.42)	1.82 (1.25, 2.58)
4	11.06.-22.06.20	4203	74.66	25.34	1.68 (1.47, 1.91)	0.58 (0.50, 0.68)	4.55 (3.73, 5.54)	1.82 (1.51, 2.16)
5	26.06.-01.07.20	2989	69.32	30.68	1.29 (1.13, 1.47)	0.59 (0.49, 0.70)	3.72 (3.07, 4.42)	1.96 (1.50, 2.50)
6	09.07.-16.07.20	2526	70.51	29.49	1.17 (0.99, 1.34)	0.50 (0.42, 0.58)	3.47 (2.76, 4.29)	1.58 (1.28, 1.89)
7	24.07.-29.07.20	1578	68.76	31.24	0.90 (0.77, 1.04)	0.43 (0.35, 0.51)	2.64 (2.17, 3.17)	1.35 (1.08, 1.70)
8	07.08.-11.08.20	1853	73.77	26.23	1.27 (1.00, 1.59)	0.46 (0.36, 0.58)	3.87 (2.67, 5.29)	1.50 (1.11, 1.94)
9	04.09.-09.09.20	1221	72.73	27.27	1.00 (0.84, 1.20)	0.39 (0.30, 0.49)	2.80 (2.21, 3.50)	1.37 (0.99, 1.86)
10	30.09.-05.10.20	1019	72.82	27.18	1.05 (0.89, 1.24)	0.42 (0.31, 0.57)	3.24 (2.52, 4.15)	1.63 (1.09, 2.39)
11	14.10.-21.10.20	1840	82.07	17.93	0.98 (0.88, 1.09)	0.23 (0.18, 0.27)	2.95 (2.51, 3.42)	0.86 (0.66, 1.09)
12	29.10.-03.11.20	2139	81.11	18.89	1.15 (0.96, 1.36)	0.29 (0.23, 0.36)	3.51 (2.63, 4.64)	1.02 (0.75, 1.36)
13	05.11.-10.11.20	1764	80.67	19.33	0.93 (0.79, 1.10)	0.23 (0.19, 0.29)	2.88 (2.28, 3.55)	0.90 (0.66, 1.20)
14	25.11.-30.11.20	1579	78.91	21.09	0.85 (0.73, 1.00)	0.24 (0.18, 0.31)	2.42 (1.82, 3.14)	0.88 (0.58, 1.28)
15	09.12.-15.12.20	1737	79.74	20.26	0.95 (0.77, 1.13)	0.28 (0.19, 0.39)	2.25 (1.70, 2.87)	1.07 (0.64, 1.61)
16	23.12.-30.12.20	1373	85.51	14.49	0.77 (0.62, 0.94)	0.13 (0.10, 0.17)	2.10 (1.57, 2.77)	0.41 (0.27, 0.58)
17	28.01.-02.02.21	976	86.58	13.42	0.85 (0.65, 1.09)	0.14 (0.08, 0.23)	2.17 (1.50, 3.08)	0.43 (0.21, 0.75)
18	24.02.-03.03.21	1678	78.61	21.39	0.90 (0.75, 1.10)	0.27 (0.19, 0.37)	2.91 (2.12, 3.81)	0.99 (0.62, 1.46)
19	17.03.-26.03.21	1942	80.95	19.05	1.08 (0.86, 1.34)	0.27 (0.19, 0.36)	3.03 (2.12, 4.09)	1.03 (0.66, 1.42)
20	07.04.-15.04.21	1340	81.04	18.96	0.71 (0.59, 0.87)	0.18 (0.13, 0.25)	1.82 (1.41, 2.28)	0.67 (0.44, 0.94)
21	12.05.-24.05.21	3703	76.15	23.85	1.18 (1.04, 1.33)	0.38 (0.30, 0.47)	3.38 (2.75, 4.11)	1.51 (1.10, 1.97)
22	26.05.-03.06.21	3370	68.40	31.60	0.97 (0.82, 1.13)	0.46 (0.39, 0.55)	2.69 (2.19, 3.31)	1.68 (1.30, 2.08)
23	09.06.-22.06.21	3866	62.13	37.87	0.98 (0.85, 1.11)	0.61 (0.50, 0.75)	3.07 (2.53, 3.63)	2.31 (1.77, 2.85)
24	07.07.-19.07.21	3416	69.73	30.27	0.97 (0.83, 1.12)	0.43 (0.36, 0.51)	2.43 (2.06, 2.84)	1.49 (1.16, 1.87)
25	04.08.-13.08.21	3918	70.04	29.96	1.12 (0.96, 1.30)	0.49 (0.40, 0.58)	2.85 (2.27, 3.61)	1.63 (1.27, 2.03)
26	01.09.-14.09.21	4304	69.91	30.09	1.22 (1.07, 1.40)	0.53 (0.45, 0.63)	3.37 (2.78, 4.04)	1.85 (1.46, 2.33)
27	22.09.-06.10.21	5663	71.55	28.45	1.63 (1.43, 1.84)	0.71 (0.57, 0.85)	4.86 (4.15, 5.64)	2.76 (2.18, 3.40)
28	08.10.-20.10.21	4562	73.35	26.65	1.35 (1.19, 1.51)	0.51 (0.41, 0.61)	4.14 (3.46, 4.86)	2.10 (1.59, 2.66)
29	22.10.-02.11.21	4739	74.15	25.85	1.43 (1.27, 1.61)	0.50 (0.40, 0.61)	4.08 (3.48, 4.72)	1.97 (1.49, 2.47)
30	03.11.-09.11.21	4102	79.62	20.38	1.33 (1.17, 1.50)	0.34 (0.27, 0.42)	3.94 (3.24, 4.68)	1.37 (1.02, 1.76)
31	17.11.-23.11.21	4430	78.26	21.74	1.40 (1.22, 1.61)	0.41 (0.32, 0.52)	4.08 (3.34, 4.89)	1.70 (1.24, 2.20)
32	08-12.-17.12.21	4367	80.10	19.90	1.40 (1.23, 1.59)	0.38 (0.29, 0.47)	3.71 (3.06, 4.39)	1.47 (1.03, 1.94)
33	24-12.-31.12.21	2748	85.92	14.08	0.93 (0.81, 1.06)	0.16 (0.12, 0.20)	2.55 (2.22, 2.87)	0.61 (0.45, 0.78)
Overall		91382	74.95	25.05	1.15 (1.12, 1.18)	0.40 (0.39, 0.42)	3.28 (3.15, 3.42)	1.46 (1.38, 1.53)

Table AF8. The percentage and bootstrap mean number of non-household contacts (with 95%CI) stratified by the participant's relationship with the contact.

			Percentage (%)				Mean number of contacts (95% CI)			
Wave	Timing	N	Work/School	Friend	Relative	Other	Work/School	Friend	Relative	Other
1	30.04.-06.05.20	912	22.81	15.35	24.34	37.50	0.14 (0.10, 0.18)	0.09 (0.07, 0.11)	0.14 (0.12, 0.18)	0.21 (0.18, 0.25)
2	14.05.-21.05.20	2991	25.68	8.79	11.23	54.30	0.55 (0.44, 0.68)	0.19 (0.16, 0.22)	0.25 (0.21, 0.29)	1.13 (0.92, 1.39)
3	28.05.-04.06.20	2291	24.62	10.13	12.35	52.90	0.55 (0.41, 0.69)	0.21 (0.17, 0.26)	0.25 (0.21, 0.30)	1.00 (0.81, 1.21)
4	11.06.-22.06.20	3942	26.20	11.95	15.93	45.92	0.57 (0.45, 0.72)	0.25 (0.21, 0.29)	0.33 (0.27, 0.40)	0.95 (0.79, 1.12)
5	26.06.-01.07.20	2657	24.05	14.75	16.94	44.26	0.43 (0.34, 0.54)	0.24 (0.20, 0.29)	0.28 (0.24, 0.32)	0.69 (0.59, 0.83)
6	09.07.-16.07.20	2315	32.96	15.29	12.61	39.14	0.50 (0.38, 0.64)	0.23 (0.18, 0.28)	0.20 (0.16, 0.23)	0.58 (0.51, 0.67)
7	24.07.-29.07.20	1450	26.41	16.48	15.10	42.00	0.32 (0.24, 0.41)	0.20 (0.16, 0.24)	0.19 (0.15, 0.23)	0.50 (0.39, 0.63)
8	07.08.-11.08.20	1656	27.84	11.90	11.71	48.55	0.42 (0.26, 0.61)	0.18 (0.14, 0.23)	0.20 (0.15, 0.24)	0.73 (0.54, 0.97)
9	04.09.-09.09.20	1055	24.83	14.88	16.97	43.32	0.31 (0.22, 0.41)	0.17 (0.13, 0.22)	0.20 (0.15, 0.24)	0.51 (0.41, 0.65)
10	30.09.-05.10.20	901	30.19	14.87	9.43	45.50	0.45 (0.32, 0.62)	0.17 (0.13, 0.21)	0.14 (0.09, 0.18)	0.48 (0.40, 0.58)
11	14.10.-21.10.20	1735	30.84	15.04	14.76	39.37	0.38 (0.30, 0.46)	0.17 (0.14, 0.20)	0.17 (0.14, 0.20)	0.41 (0.36, 0.47)
12	29.10.-03.11.20	1954	28.66	12.90	13.66	44.78	0.39 (0.30, 0.51)	0.18 (0.13, 0.23)	0.19 (0.12, 0.29)	0.53 (0.41, 0.70)
13	05.11.-10.11.20	1612	38.21	10.42	12.47	38.90	0.43 (0.31, 0.56)	0.11 (0.09, 0.14)	0.13 (0.11, 0.16)	0.38 (0.31, 0.49)
14	25.11.-30.11.20	1375	32.36	8.07	14.40	45.16	0.31 (0.24, 0.41)	0.07 (0.06, 0.09)	0.13 (0.10, 0.17)	0.41 (0.33, 0.51)
15	09.12.-15.12.20	1568	35.40	6.63	11.73	46.24	0.41 (0.30, 0.53)	0.07 (0.05, 0.09)	0.12 (0.10, 0.16)	0.47 (0.35, 0.61)
16	23.12.-30.12.20	1333	15.53	7.88	29.86	46.74	0.14 (0.09, 0.19)	0.07 (0.05, 0.09)	0.27 (0.22, 0.31)	0.39 (0.27, 0.57)
17	28.01.-02.02.21	904	33.85	7.74	12.39	46.02	0.34 (0.21, 0.50)	0.07 (0.05, 0.09)	0.11 (0.08, 0.15)	0.39 (0.26, 0.60)
18	24.02.-03.03.21	1450	32.69	9.59	13.31	44.41	0.36 (0.24, 0.48)	0.09 (0.07, 0.11)	0.13 (0.10, 0.17)	0.41 (0.30, 0.55)
19	17.03.-26.03.21	1689	35.46	5.68	10.60	48.25	0.44 (0.32, 0.57)	0.07 (0.05, 0.08)	0.13 (0.09, 0.17)	0.53 (0.36, 0.74)
20	07.04.-15.04.21	1263	21.46	9.03	15.60	53.92	0.19 (0.13, 0.27)	0.08 (0.06, 0.10)	0.13 (0.10, 0.16)	0.43 (0.33, 0.56)
21	12.05.-24.05.21	3274	36.68	9.74	13.07	40.50	0.51 (0.42, 0.61)	0.13 (0.10, 0.16)	0.18 (0.15, 0.21)	0.55 (0.45, 0.66)
22	26.05.-03.06.21	2931	28.15	15.05	13.95	42.85	0.36 (0.28, 0.45)	0.18 (0.14, 0.22)	0.16 (0.14, 0.19)	0.52 (0.41, 0.64)
23	09.06.-22.06.21	3158	37.33	15.07	11.84	35.75	0.50 (0.39, 0.61)	0.19 (0.16, 0.24)	0.15 (0.12, 0.18)	0.44 (0.36, 0.52)
24	07.07.-19.07.21	2852	26.65	12.94	15.74	44.67	0.31 (0.24, 0.38)	0.15 (0.12, 0.18)	0.18 (0.15, 0.22)	0.51 (0.40, 0.63)
25	04.08.-13.08.21	3326	26.55	12.72	16.33	44.41	0.38 (0.30, 0.46)	0.17 (0.14, 0.20)	0.21 (0.19, 0.24)	0.59 (0.46, 0.74)
26	01.09.-14.09.21	3695	29.88	13.99	16.24	39.89	0.47 (0.38, 0.57)	0.21 (0.18, 0.25)	0.24 (0.19, 0.31)	0.58 (0.48, 0.69)
27	22.09.-06.10.21	4764	37.24	9.24	8.42	45.11	0.78 (0.65, 0.91)	0.18 (0.15, 0.22)	0.15 (0.13, 0.17)	0.80 (0.65, 0.98)
28	08.10.-20.10.21	3839	36.16	11.72	11.15	40.97	0.61 (0.51, 0.72)	0.17 (0.13, 0.21)	0.16 (0.14, 0.19)	0.60 (0.50, 0.70)
29	22.10.-02.11.21	4031	29.67	13.67	13.40	43.26	0.50 (0.41, 0.60)	0.22 (0.19, 0.27)	0.22 (0.18, 0.26)	0.69 (0.58, 0.82)
30	03.11.-09.11.21	3646	35.19	12.34	15.06	37.41	0.55 (0.44, 0.69)	0.18 (0.15, 0.23)	0.22 (0.18, 0.27)	0.53 (0.44, 0.63)
31	17.11.-23.11.21	3759	38.33	9.07	10.43	42.17	0.62 (0.49, 0.74)	0.14 (0.11, 0.17)	0.16 (0.13, 0.18)	0.61 (0.48, 0.75)
32	08-12.-17.12.21	3794	34.16	8.83	11.60	45.41	0.56 (0.46, 0.67)	0.13 (0.10, 0.17)	0.18 (0.14, 0.21)	0.65 (0.53, 0.80)
33	24-12.-31.12.21	2525	14.22	10.69	40.20	34.89	0.15 (0.11, 0.19)	0.11 (0.09, 0.13)	0.40 (0.35, 0.45)	0.34 (0.25, 0.45)
Overall		80647	30.52	11.63	14.43	43.42	0.44 (0.42, 0.46)	0.16 (0.15, 0.16)	0.19 (0.19, 0.20)	0.56 (0.54, 0.59)

Table AF9. The bootstrap mean duration (hours) of non-household contacts (with 95% CI) stratified by by the participant's relationship with the contact.

		Mean duration of contacts in hours (95% CI)			
**Wave**	**Timing**	**Work/School**	**Friend**	**Relative**	**Other**
1	30.04.-06.05.20	0.40 (0.29, 0.53)	0.26 (0.20, 0.32)	0.42 (0.31, 0.55)	0.23 (0.18, 0.29)
2	14.05.-21.05.20	1.98 (1.52, 2.48)	0.52 (0.43, 0.63)	0.68 (0.56, 0.82)	2.54 (1.55, 3.90)
3	28.05.-04.06.20	2.24 (1.58, 2.96)	0.61 (0.48, 0.76)	0.76 (0.59, 0.98)	1.76 (1.05, 2.60)
4	11.06.-22.06.20	2.09 (1.49, 2.90)	0.76 (0.62, 0.89)	1.07 (0.82, 1.41)	1.52 (1.05, 2.11)
5	26.06.-01.07.20	1.69 (1.23, 2.28)	0.73 (0.59, 0.88)	0.89 (0.73, 1.05)	1.07 (0.75, 1.45)
6	09.07.-16.07.20	2.14 (1.49, 2.95)	0.70 (0.54, 0.87)	0.61 (0.49, 0.74)	0.72 (0.53, 0.97)
7	24.07.-29.07.20	1.36 (0.97, 1.80)	0.61 (0.48, 0.75)	0.66 (0.49, 0.85)	0.62 (0.41, 0.93)
8	07.08.-11.08.20	1.61 (1.02, 2.29)	0.55 (0.37, 0.75)	0.65 (0.49, 0.84)	1.57 (0.74, 2.80)
9	04.09.-09.09.20	1.19 (0.81, 1.68)	0.53 (0.36, 0.71)	0.62 (0.46, 0.79)	0.73 (0.46, 1.10)
10	30.09.-05.10.20	2.03 (1.33, 2.88)	0.48 (0.35, 0.60)	0.37 (0.23, 0.53)	0.70 (0.43, 1.02)
11	14.10.-21.10.20	1.58 (1.21, 2.02)	0.52 (0.41, 0.65)	0.56 (0.43, 0.68)	0.54 (0.40, 0.70)
12	29.10.-03.11.20	1.65 (1.14, 2.29)	0.51 (0.38, 0.64)	0.54 (0.36, 0.81)	1.06 (0.44, 2.04)
13	05.11.-10.11.20	1.74 (1.23, 2.27)	0.35 (0.27, 0.45)	0.40 (0.32, 0.49)	0.60 (0.34, 0.96)
14	25.11.-30.11.20	1.14 (0.78, 1.62)	0.18 (0.12, 0.23)	0.37 (0.26, 0.50)	0.83 (0.47, 1.27)
15	09.12.-15.12.20	1.39 (0.95, 1.90)	0.14 (0.10, 0.19)	0.30 (0.22, 0.40)	0.55 (0.34, 0.86)
16	23.12.-30.12.20	0.56 (0.33, 0.82)	0.14 (0.11, 0.19)	0.93 (0.74, 1.13)	0.58 (0.22, 1.17)
17	28.01.-02.02.21	1.49 (0.85, 2.36)	0.17 (0.11, 0.25)	0.24 (0.15, 0.32)	0.32 (0.22, 0.44)
18	24.02.-03.03.21	1.45 (0.87, 2.09)	0.21 (0.16, 0.26)	0.39 (0.24, 0.59)	1.01 (0.52, 1.64)
19	17.03.-26.03.21	1.83 (1.25, 2.46)	0.17 (0.12, 0.22)	0.32 (0.23, 0.42)	0.79 (0.34, 1.47)
20	07.04.-15.04.21	0.74 (0.48, 1.08)	0.20 (0.14, 0.27)	0.41 (0.31, 0.53)	0.57 (0.34, 0.85)
21	12.05.-24.05.21	1.82 (1.35, 2.34)	0.30 (0.21, 0.41)	0.45 (0.36, 0.54)	1.08 (0.69, 1.61)
22	26.05.-03.06.21	1.45 (1.08, 1.87)	0.44 (0.34, 0.56)	0.44 (0.35, 0.54)	0.81 (0.56, 1.13)
23	09.06.-22.06.21	2.07 (1.56, 2.58)	0.60 (0.43, 0.81)	0.39 (0.31, 0.50)	0.71 (0.50, 0.95)
24	07.07.-19.07.21	1.17 (0.85, 1.50)	0.40 (0.31, 0.50)	0.56 (0.42, 0.77)	0.68 (0.49, 0.89)
25	04.08.-13.08.21	1.24 (0.91, 1.64)	0.46 (0.38, 0.56)	0.60 (0.50, 0.71)	0.98 (0.55, 1.58)
26	01.09.-14.09.21	1.68 (1.23, 2.15)	0.60 (0.46, 0.77)	0.61 (0.51, 0.72)	1.07 (0.72, 1.49)
27	22.09.-06.10.21	3.21 (2.57, 3.90)	0.45 (0.33, 0.58)	0.39 (0.31, 0.48)	1.24 (0.93, 1.63)
28	08.10.-20.10.21	2.35 (1.84, 2.93)	0.54 (0.38, 0.73)	0.46 (0.37, 0.55)	1.16 (0.83, 1.52)
29	22.10.-02.11.21	1.89 (1.46, 2.35)	0.69 (0.53, 0.89)	0.58 (0.47, 0.69)	1.26 (0.88, 1.71)
30	03.11.-09.11.21	2.14 (1.59, 2.77)	0.48 (0.36, 0.64)	0.61 (0.45, 0.85)	0.88 (0.62, 1.18)
31	17.11.-23.11.21	2.52 (1.91, 3.22)	0.39 (0.28, 0.53)	0.39 (0.31, 0.47)	0.93 (0.62, 1.36)
32	08-12.-17.12.21	2.01 (1.54, 2.46)	0.36 (0.24, 0.53)	0.44 (0.32, 0.60)	1.02 (0.64, 1.51)
33	24-12.-31.12.21	0.35 (0.24, 0.50)	0.30 (0.24, 0.36)	1.51 (1.31, 1.71)	0.43 (0.24, 0.66)
Overall		1.68 (1.59, 1.78)	0.44 (0.42, 0.46)	0.57 (0.55, 0.60)	0.93 (0.85, 1.02)

Table AF10. The percentage of non-household contacts (with 95% CI) stratified by the frequency of contact before the SARS-CoV-2 pandemic.

			Percentage (%)						
**Wave**	**Timing**	**N**	**Never**	**Less 1/month**	**One/month**	**Every 2-3 wks**	**1or2 a week**	**Almost (daily)**	**Missing**
1	30.04.-06.05.20	912	9.21	10.31	5.70	11.29	32.24	30.48	0.77
2	14.05.-21.05.20	2991	10.57	6.69	4.75	8.99	24.81	42.16	2.04
3	28.05.-04.06.20	2291	12.88	8.12	5.19	7.77	27.15	36.23	2.66
4	11.06.-22.06.20	3942	12.63	7.71	4.69	8.63	27.83	36.68	1.83
5	26.06.-01.07.20	2657	14.45	8.66	5.98	9.15	27.32	32.52	1.92
6	09.07.-16.07.20	2315	11.06	7.30	5.87	9.03	27.26	36.67	2.81
7	24.07.-29.07.20	1450	15.31	10.07	5.38	8.69	23.86	35.59	1.10
8	07.08.-11.08.20	1656	19.69	10.39	4.05	7.97	21.07	36.59	0.24
9	04.09.-09.09.20	1055	14.12	10.71	5.69	11.00	26.45	29.67	2.37
10	30.09.-05.10.20	901	12.43	8.77	4.33	7.33	29.97	34.85	2.33
11	14.10.-21.10.20	1735	12.39	9.74	5.13	9.80	27.03	34.87	1.04
12	29.10.-03.11.20	1954	14.02	5.94	4.15	6.96	27.12	40.12	1.69
13	05.11.-10.11.20	1612	11.41	8.44	3.91	7.44	22.70	45.04	1.05
14	25.11.-30.11.20	1375	10.91	6.55	3.85	8.44	25.45	44.51	0.29
15	09.12.-15.12.20	1568	15.31	5.42	2.68	5.87	20.98	48.53	1.21
16	23.12.-30.12.20	1333	12.68	16.50	7.73	12.75	22.51	27.31	0.53
17	28.01.-02.02.21	904	19.36	6.19	2.65	7.74	19.36	43.69	1.00
18	24.02.-03.03.21	1450	11.03	7.52	3.10	5.03	21.31	50.48	1.52
19	17.03.-26.03.21	1689	17.23	7.16	2.07	4.68	19.01	49.14	0.71
20	07.04.-15.04.21	1263	14.96	9.26	4.20	8.47	22.49	39.35	1.27
21	12.05.-24.05.21	3274	11.82	7.03	4.09	6.17	27.18	41.57	2.14
22	26.05.-03.06.21	2931	12.32	10.17	4.81	7.47	25.08	38.11	2.05
23	09.06.-22.06.21	3158	8.64	7.60	4.24	6.93	28.44	40.22	3.93
24	07.07.-19.07.21	2852	12.27	11.71	3.96	5.89	22.62	41.73	1.82
25	04.08.-13.08.21	3326	12.48	12.39	4.75	8.27	23.42	35.96	2.74
26	01.09.-14.09.21	3695	10.72	9.47	5.44	8.93	23.82	38.78	2.84
27	22.09.-06.10.21	4764	12.85	5.37	3.06	5.63	21.01	49.14	2.94
28	08.10.-20.10.21	3839	10.50	7.16	3.88	5.55	27.79	42.22	2.89
29	22.10.-02.11.21	4031	12.01	8.61	4.17	7.54	24.01	41.63	2.03
30	03.11.-09.11.21	3646	12.31	8.01	6.39	7.57	22.44	40.59	2.69
31	17.11.-23.11.21	3759	8.49	5.67	4.15	5.53	22.40	50.84	2.93
32	08-12.-17.12.21	3794	10.49	7.33	2.90	6.77	24.88	46.81	0.82
33	24-12.-31.12.21	2525	7.25	18.14	8.95	10.50	30.42	23.60	1.15
Overall		80647	12.05	8.55	4.58	7.59	24.83	40.36	0.00

Table AF11. The bootstrap mean number of non-household contacts (with 95% CI) stratified by the frequency of contact before the SARS-CoV-2 pandemic.

		Mean number of contacts (95% CI)					
**Wave**	**Timing**	**Never**	**Less 1/month**	**One/month**	**Every 2-3 wks**	**1or2 a week**	**Almost (daily)**
1	30.04.-06.05.20	0.05 (0.04, 0.08)	0.06 (0.04, 0.08)	0.03 (0.02, 0.05)	0.07 (0.05, 0.09)	0.18 (0.15, 0.22)	0.17 (0.14, 0.21)
2	14.05.-21.05.20	0.22 (0.17, 0.28)	0.15 (0.12, 0.19)	0.10 (0.08, 0.13)	0.20 (0.15, 0.28)	0.52 (0.43, 0.62)	0.88 (0.67, 1.15)
3	28.05.-04.06.20	0.24 (0.17, 0.31)	0.16 (0.11, 0.21)	0.10 (0.08, 0.13)	0.15 (0.12, 0.19)	0.52 (0.42, 0.64)	0.77 (0.58, 0.98)
4	11.06.-22.06.20	0.27 (0.20, 0.35)	0.16 (0.13, 0.19)	0.09 (0.07, 0.11)	0.17 (0.14, 0.22)	0.57 (0.49, 0.66)	0.80 (0.63, 0.99)
5	26.06.-01.07.20	0.22 (0.16, 0.29)	0.14 (0.11, 0.18)	0.10 (0.06, 0.15)	0.15 (0.12, 0.17)	0.43 (0.37, 0.49)	0.58 (0.47, 0.70)
6	09.07.-16.07.20	0.17 (0.13, 0.21)	0.11 (0.09, 0.13)	0.09 (0.07, 0.12)	0.14 (0.11, 0.16)	0.40 (0.34, 0.47)	0.56 (0.42, 0.70)
7	24.07.-29.07.20	0.19 (0.14, 0.24)	0.12 (0.10, 0.15)	0.07 (0.05, 0.10)	0.11 (0.08, 0.14)	0.29 (0.24, 0.33)	0.42 (0.31, 0.55)
8	07.08.-11.08.20	0.30 (0.19, 0.42)	0.16 (0.11, 0.22)	0.06 (0.04, 0.08)	0.13 (0.09, 0.16)	0.33 (0.25, 0.41)	0.56 (0.35, 0.82)
9	04.09.-09.09.20	0.17 (0.10, 0.28)	0.13 (0.09, 0.17)	0.07 (0.04, 0.09)	0.12 (0.09, 0.16)	0.30 (0.25, 0.36)	0.37 (0.28, 0.48)
10	30.09.-05.10.20	0.14 (0.10, 0.19)	0.10 (0.06, 0.13)	0.05 (0.03, 0.07)	0.08 (0.05, 0.11)	0.32 (0.26, 0.39)	0.51 (0.38, 0.68)
11	14.10.-21.10.20	0.14 (0.11, 0.17)	0.11 (0.08, 0.14)	0.06 (0.04, 0.07)	0.10 (0.08, 0.12)	0.29 (0.25, 0.33)	0.42 (0.35, 0.50)
12	29.10.-03.11.20	0.17 (0.14, 0.21)	0.07 (0.06, 0.10)	0.05 (0.04, 0.07)	0.09 (0.07, 0.11)	0.35 (0.26, 0.46)	0.53 (0.39, 0.71)
13	05.11.-10.11.20	0.12 (0.08, 0.17)	0.08 (0.06, 0.10)	0.04 (0.03, 0.06)	0.07 (0.06, 0.09)	0.23 (0.19, 0.28)	0.50 (0.38, 0.65)
14	25.11.-30.11.20	0.09 (0.07, 0.13)	0.06 (0.04, 0.08)	0.03 (0.02, 0.05)	0.07 (0.05, 0.10)	0.23 (0.19, 0.28)	0.43 (0.33, 0.56)
15	09.12.-15.12.20	0.15 (0.08, 0.25)	0.05 (0.04, 0.07)	0.03 (0.02, 0.04)	0.06 (0.04, 0.08)	0.22 (0.18, 0.26)	0.55 (0.41, 0.70)
16	23.12.-30.12.20	0.10 (0.03, 0.23)	0.14 (0.10, 0.19)	0.07 (0.05, 0.09)	0.11 (0.08, 0.17)	0.19 (0.16, 0.23)	0.24 (0.17, 0.36)
17	28.01.-02.02.21	0.16 (0.07, 0.35)	0.05 (0.04, 0.07)	0.02 (0.01, 0.04)	0.07 (0.05, 0.09)	0.17 (0.14, 0.21)	0.42 (0.28, 0.60)
18	24.02.-03.03.21	0.10 (0.06, 0.17)	0.07 (0.04, 0.09)	0.03 (0.02, 0.04)	0.05 (0.04, 0.06)	0.20 (0.16, 0.24)	0.53 (0.38, 0.69)
19	17.03.-26.03.21	0.18 (0.08, 0.34)	0.08 (0.04, 0.14)	0.02 (0.01, 0.03)	0.05 (0.04, 0.07)	0.21 (0.16, 0.26)	0.61 (0.42, 0.82)
20	07.04.-15.04.21	0.12 (0.08, 0.18)	0.07 (0.05, 0.09)	0.03 (0.02, 0.05)	0.07 (0.05, 0.09)	0.19 (0.15, 0.24)	0.33 (0.23, 0.46)
21	12.05.-24.05.21	0.16 (0.11, 0.21)	0.09 (0.06, 0.14)	0.05 (0.04, 0.07)	0.08 (0.07, 0.10)	0.37 (0.31, 0.43)	0.59 (0.47, 0.71)
22	26.05.-03.06.21	0.14 (0.10, 0.19)	0.13 (0.09, 0.18)	0.06 (0.04, 0.09)	0.09 (0.07, 0.10)	0.30 (0.25, 0.35)	0.49 (0.38, 0.61)
23	09.06.-22.06.21	0.11 (0.08, 0.14)	0.09 (0.07, 0.11)	0.05 (0.04, 0.07)	0.08 (0.07, 0.11)	0.35 (0.28, 0.43)	0.54 (0.43, 0.66)
24	07.07.-19.07.21	0.14 (0.10, 0.19)	0.13 (0.10, 0.18)	0.04 (0.03, 0.06)	0.06 (0.05, 0.08)	0.25 (0.22, 0.29)	0.50 (0.38, 0.63)
25	04.08.-13.08.21	0.17 (0.11, 0.23)	0.16 (0.10, 0.26)	0.06 (0.05, 0.07)	0.11 (0.08, 0.14)	0.31 (0.26, 0.35)	0.51 (0.39, 0.64)
26	01.09.-14.09.21	0.16 (0.11, 0.21)	0.13 (0.11, 0.16)	0.08 (0.05, 0.13)	0.14 (0.09, 0.20)	0.35 (0.29, 0.40)	0.60 (0.49, 0.72)
27	22.09.-06.10.21	0.22 (0.14, 0.32)	0.09 (0.07, 0.11)	0.05 (0.04, 0.07)	0.10 (0.09, 0.12)	0.39 (0.32, 0.46)	0.98 (0.83, 1.15)
28	08.10.-20.10.21	0.16 (0.12, 0.22)	0.10 (0.08, 0.13)	0.05 (0.04, 0.08)	0.08 (0.07, 0.10)	0.40 (0.33, 0.47)	0.69 (0.57, 0.83)
29	22.10.-02.11.21	0.19 (0.14, 0.24)	0.14 (0.11, 0.18)	0.07 (0.05, 0.08)	0.12 (0.10, 0.15)	0.39 (0.33, 0.46)	0.69 (0.56, 0.85)
30	03.11.-09.11.21	0.17 (0.12, 0.25)	0.11 (0.09, 0.14)	0.10 (0.06, 0.18)	0.11 (0.08, 0.14)	0.33 (0.28, 0.39)	0.62 (0.51, 0.74)
31	17.11.-23.11.21	0.13 (0.10, 0.16)	0.08 (0.06, 0.10)	0.06 (0.04, 0.08)	0.09 (0.07, 0.10)	0.33 (0.26, 0.42)	0.78 (0.63, 0.97)
32	08-12.-17.12.21	0.16 (0.10, 0.24)	0.11 (0.08, 0.14)	0.04 (0.03, 0.05)	0.10 (0.08, 0.12)	0.36 (0.29, 0.42)	0.74 (0.60, 0.90)
33	24-12.-31.12.21	0.07 (0.06, 0.09)	0.18 (0.15, 0.23)	0.09 (0.07, 0.11)	0.10 (0.09, 0.12)	0.30 (0.24, 0.36)	0.24 (0.16, 0.36)
Overall		0.16 (0.15, 0.17)	0.11 (0.11, 0.12)	0.06 (0.06, 0.07)	0.10 (0.10, 0.11)	0.33 (0.31, 0.34)	0.57 (0.54, 0.59)

Table AF12. The bootstrap mean duration (hours) of non-household contacts (with 95%CI) stratified by the frequency of contact before the SARS-CoV-2 pandemic.

		Mean duration of contacts in hours (95% CI)					
**Wave**	**Timing**	**Never**	**Less 1/month**	**One/month**	**Every 2-3 wks**	**1or2 a week**	**Almost (daily)**
1	30.04.-06.05.20	0.05 (0.02, 0.08)	0.08 (0.05, 0.12)	0.07 (0.04, 0.11)	0.16 (0.09, 0.23)	0.40 (0.30, 0.51)	0.53 (0.43, 0.65)
2	14.05.-21.05.20	0.20 (0.09, 0.35)	0.20 (0.12, 0.29)	0.17 (0.11, 0.24)	0.50 (0.25, 0.93)	1.02 (0.80, 1.28)	3.55 (2.49, 4.98)
3	28.05.-04.06.20	0.16 (0.09, 0.23)	0.30 (0.19, 0.44)	0.17 (0.11, 0.23)	0.29 (0.19, 0.44)	0.94 (0.73, 1.15)	3.20 (2.34, 4.15)
4	11.06.-22.06.20	0.28 (0.13, 0.52)	0.34 (0.25, 0.44)	0.19 (0.13, 0.25)	0.37 (0.27, 0.49)	1.19 (0.97, 1.43)	2.98 (2.24, 3.90)
5	26.06.-01.07.20	0.27 (0.14, 0.44)	0.30 (0.21, 0.40)	0.19 (0.12, 0.26)	0.32 (0.25, 0.40)	0.91 (0.75, 1.08)	2.34 (1.76, 2.96)
6	09.07.-16.07.20	0.16 (0.09, 0.28)	0.23 (0.16, 0.32)	0.17 (0.11, 0.24)	0.30 (0.21, 0.41)	0.93 (0.74, 1.16)	2.29 (1.64, 3.07)
7	24.07.-29.07.20	0.18 (0.11, 0.26)	0.24 (0.17, 0.33)	0.18 (0.11, 0.26)	0.28 (0.19, 0.39)	0.56 (0.46, 0.68)	1.77 (1.27, 2.28)
8	07.08.-11.08.20	0.42 (0.19, 0.74)	0.35 (0.22, 0.52)	0.13 (0.07, 0.21)	0.28 (0.18, 0.40)	0.91 (0.56, 1.37)	2.27 (1.36, 3.55)
9	04.09.-09.09.20	0.17 (0.07, 0.33)	0.24 (0.15, 0.36)	0.17 (0.09, 0.27)	0.28 (0.17, 0.43)	0.55 (0.42, 0.68)	1.55 (1.10, 2.11)
10	30.09.-05.10.20	0.13 (0.06, 0.21)	0.18 (0.09, 0.28)	0.07 (0.02, 0.13)	0.11 (0.06, 0.16)	0.66 (0.49, 0.84)	2.36 (1.68, 3.24)
11	14.10.-21.10.20	0.17 (0.10, 0.24)	0.21 (0.14, 0.29)	0.10 (0.06, 0.15)	0.24 (0.16, 0.33)	0.64 (0.51, 0.79)	1.81 (1.44, 2.24)
12	29.10.-03.11.20	0.22 (0.12, 0.35)	0.12 (0.08, 0.19)	0.10 (0.06, 0.14)	0.18 (0.12, 0.26)	0.80 (0.52, 1.16)	2.30 (1.49, 3.35)
13	05.11.-10.11.20	0.24 (0.08, 0.49)	0.12 (0.08, 0.17)	0.09 (0.05, 0.14)	0.15 (0.10, 0.21)	0.40 (0.32, 0.48)	2.07 (1.55, 2.67)
14	25.11.-30.11.20	0.11 (0.03, 0.25)	0.07 (0.04, 0.10)	0.06 (0.03, 0.10)	0.10 (0.06, 0.14)	0.41 (0.29, 0.54)	1.77 (1.22, 2.50)
15	09.12.-15.12.20	0.11 (0.04, 0.23)	0.04 (0.03, 0.06)	0.04 (0.02, 0.06)	0.08 (0.04, 0.12)	0.30 (0.24, 0.36)	1.77 (1.25, 2.39)
16	23.12.-30.12.20	0.03 (0.01, 0.05)	0.41 (0.24, 0.63)	0.16 (0.10, 0.23)	0.21 (0.13, 0.29)	0.38 (0.29, 0.47)	1.03 (0.59, 1.65)
17	28.01.-02.02.21	0.12 (0.02, 0.35)	0.05 (0.02, 0.08)	0.02 (0.01, 0.04)	0.09 (0.05, 0.15)	0.32 (0.22, 0.43)	1.61 (0.96, 2.45)
18	24.02.-03.03.21	0.24 (0.03, 0.57)	0.10 (0.05, 0.16)	0.06 (0.02, 0.09)	0.09 (0.06, 0.13)	0.34 (0.26, 0.44)	2.21 (1.50, 3.04)
19	17.03.-26.03.21	0.07 (0.03, 0.13)	0.09 (0.05, 0.14)	0.03 (0.01, 0.05)	0.10 (0.05, 0.16)	0.41 (0.27, 0.58)	2.39 (1.46, 3.47)
20	07.04.-15.04.21	0.13 (0.04, 0.30)	0.16 (0.10, 0.23)	0.03 (0.02, 0.05)	0.13 (0.08, 0.18)	0.36 (0.26, 0.48)	1.08 (0.75, 1.47)
21	12.05.-24.05.21	0.16 (0.07, 0.28)	0.23 (0.10, 0.47)	0.08 (0.05, 0.11)	0.15 (0.11, 0.20)	0.66 (0.49, 0.86)	2.29 (1.71, 2.92)
22	26.05.-03.06.21	0.16 (0.08, 0.24)	0.34 (0.17, 0.57)	0.06 (0.04, 0.09)	0.18 (0.13, 0.24)	0.65 (0.48, 0.83)	1.66 (1.25, 2.09)
23	09.06.-22.06.21	0.17 (0.08, 0.29)	0.20 (0.13, 0.28)	0.10 (0.06, 0.13)	0.18 (0.12, 0.25)	0.80 (0.59, 1.06)	2.16 (1.66, 2.64)
24	07.07.-19.07.21	0.20 (0.08, 0.41)	0.28 (0.20, 0.39)	0.10 (0.06, 0.15)	0.12 (0.08, 0.17)	0.45 (0.38, 0.54)	1.56 (1.20, 1.94)
25	04.08.-13.08.21	0.22 (0.12, 0.35)	0.53 (0.22, 1.08)	0.11 (0.08, 0.15)	0.23 (0.16, 0.32)	0.64 (0.50, 0.80)	1.52 (1.13, 1.91)
26	01.09.-14.09.21	0.26 (0.13, 0.48)	0.25 (0.19, 0.32)	0.13 (0.09, 0.18)	0.28 (0.21, 0.35)	0.62 (0.51, 0.74)	2.27 (1.73, 2.90)
27	22.09.-06.10.21	0.25 (0.14, 0.38)	0.13 (0.08, 0.20)	0.10 (0.05, 0.17)	0.18 (0.13, 0.23)	0.69 (0.56, 0.84)	3.76 (3.03, 4.49)
28	08.10.-20.10.21	0.29 (0.17, 0.42)	0.22 (0.15, 0.29)	0.11 (0.07, 0.16)	0.17 (0.10, 0.25)	0.80 (0.59, 1.05)	2.73 (2.12, 3.38)
29	22.10.-02.11.21	0.24 (0.16, 0.34)	0.38 (0.27, 0.52)	0.15 (0.10, 0.21)	0.23 (0.17, 0.31)	0.85 (0.61, 1.14)	2.50 (1.96, 3.11)
30	03.11.-09.11.21	0.37 (0.16, 0.73)	0.23 (0.16, 0.30)	0.30 (0.10, 0.75)	0.19 (0.13, 0.26)	0.70 (0.51, 0.96)	2.28 (1.78, 2.80)
31	17.11.-23.11.21	0.17 (0.12, 0.24)	0.13 (0.08, 0.19)	0.10 (0.07, 0.14)	0.17 (0.12, 0.22)	0.57 (0.42, 0.84)	2.92 (2.24, 3.69)
32	08-12.-17.12.21	0.37 (0.09, 0.83)	0.17 (0.10, 0.26)	0.05 (0.03, 0.08)	0.15 (0.10, 0.20)	0.53 (0.41, 0.69)	2.51 (1.96, 3.06)
33	24-12.-31.12.21	0.08 (0.05, 0.12)	0.64 (0.49, 0.79)	0.27 (0.21, 0.35)	0.28 (0.22, 0.35)	0.62 (0.51, 0.75)	0.68 (0.47, 0.94)
Overall		0.20 (0.17, 0.24)	0.24 (0.22, 0.28)	0.12 (0.11, 0.14)	0.20 (0.19, 0.22)	0.64 (0.61, 0.68)	2.14 (2.02, 2.26)

IV. Weighting

Table AF13: The percentage of demographic characteristics used for weighting, including day of the week, gender, household size, age group, and federal state, between the projected demographic data of Germany in 2019 (census) and the COVIMOD study (survey).

Day of a week			Gender			Household size			Age group			Federal state		
	census	survey		census	survey		census	survey		census	survey		census	survey
**weekday**	71.43	78.76	**Female**	50.54	47.49	**1**	20.05	28.26	**0-9**	9.24	6.72	**Baden-Württemberg**	13.35	12.84
**weekend**	28.57	21.24	**Male**	49.46	52.51	**2**	33.53	33.97	**10-19**	9.19	9.29	**Bayern**	15.78	15.91
						**3**	17.96	21.4	**20-34**	18.25	16.1	**Berlin**	4.41	4.49
						**4**	19.3	10.49	**35-44**	12.26	9.44	**Brandenburg**	3.03	2.90
						**5+**	9.17	5.87	**45-54**	14.41	14.91	**Bremen**	0.82	0.79
									**55-64**	14.89	16.89	**Hamburg**	2.22	2.67
									**65+**	21.75	26.65	**Hessen**	7.56	8.14
												**Mecklenburg-Vorpommern**	1.93	1.87
												**Niedersachsen**	9.61	8.39
												**Nordrhein-Westfalen**	21.58	21.67
												**Rheinland-Pfalz**	4.92	4.65
												**Saarland**	1.19	1.00
												**Sachsen**	4.90	4.01
												**Sachsen-Anhalt**	2.64	3.07
												**Schleswig-Holstein**	3.49	4.38
												**Thüringen**	2.57	3.20

V. Settings definition

For each contact reported, we also asked where the contact happened. The settings asked for were at work, at school (including contacts at childcare, schools, and universities), at home, at somebody else's home, at a place of worship, at a shop for non-essential items, at a place of entertainment (e.g. bar, restaurant, cinema), at a place of sport (e.g. gym, sports club), outside (e.g. at a park), at a beauty place (e.g. hairdresser, nail salon), at transport, at healthcare setting (e.g. hospital, GP, dentist), at a shop for essential goods, and other settings.

We regrouped these settings into 4 main categories for analysis, as shown in the table below.

Table AF14. The reported settings where the contacts happened in the COVIMOD study**.**

**Reported settings**	**Regrouped settings**
Work	Work
School	School
Home own	Home/Leisure
Home else	
Shop non-essential	
Entertainment	
Sport	
Park	
Beauty	
Worship	
Transport	Other
Healthcare	
Shop essential	
Somewhere else	
Not specified	

VI. Social contacts with further stratification

1. Age group

Figure AF3. Social contact variation stratified by age groups between April 2020 and December 2021 in Germany.

(A) Mean number of contacts per person per day; (B) Mean cumulative duration of contacts per person per day (in hours). For comparison, the stringency of the NPIs in Germany (OxCGRT) over the study period is provided in grey shadow on the right y-axis.

The solid line represents the mean number of contacts; the shaded area illustrates the 95% CI obtained using bootstrapping.

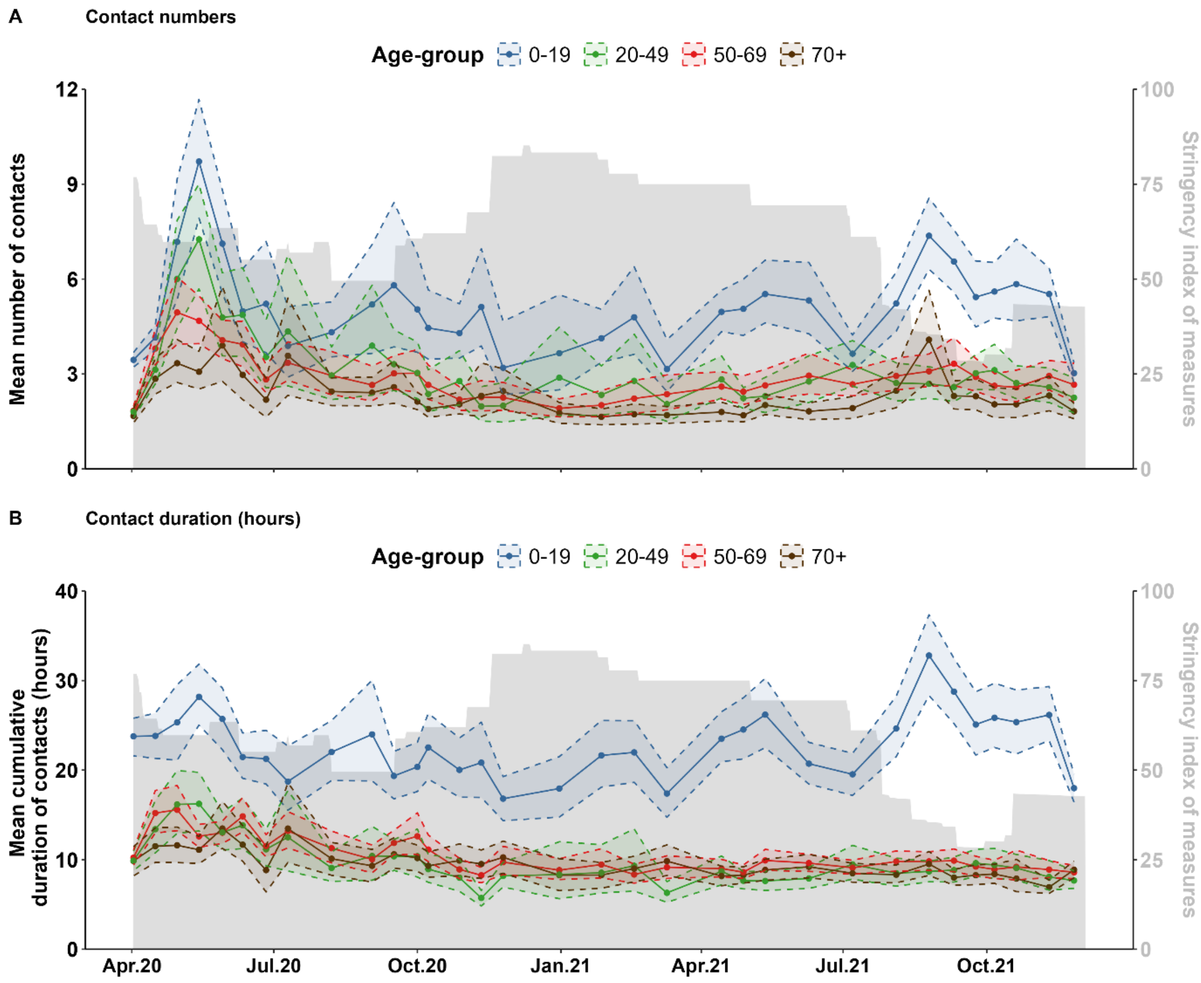

Table AF15. The bootstrapped mean number of contacts and accumulative duration of contacts (hours) with 95%CI across 33 COVIMOD survey waves, stratified by age groups.

		Mean number of contacts (95%CI)				Mean duration of contacts in hours (95%CI)			
Wave	Timing	0-19	20-49	50-69	70+	0-19	20-49	50-69	70+
1	30.04.-06.05.20	3.45 (3.22, 3.68)	1.81 (1.61, 1.99)	1.83 (1.71, 1.97)	1.67 (1.45, 1.90)	23.77 (21.58, 25.79)	9.90 (8.77, 10.97)	10.21 (9.40, 11.09)	9.86 (8.19, 11.45)
2	29.10.-03.11.20	4.15 (3.76, 4.55)	3.13 (2.69, 3.66)	3.80 (3.30, 4.42)	2.85 (2.36, 3.48)	23.82 (21.31, 26.36)	13.40 (10.76, 16.60)	15.21 (12.97, 17.72)	11.50 (9.70, 13.55)
3	09.06.-22.06.21	7.17 (5.35, 9.16)	6.00 (4.45, 7.86)	4.94 (3.94, 6.07)	3.34 (2.73, 4.09)	25.33 (21.10, 29.53)	16.17 (12.93, 19.95)	15.57 (13.24, 18.28)	11.60 (9.68, 13.64)
4	08.10.-20.10.21	9.72 (7.93, 11.68)	7.26 (5.69, 9.00)	4.68 (3.96, 5.46)	3.07 (2.52, 3.72)	28.18 (25.03, 31.83)	16.24 (13.21, 19.75)	12.60 (11.39, 13.94)	11.10 (9.56, 12.60)
5	22.10.-02.11.21	7.12 (5.54, 8.81)	4.78 (3.57, 6.19)	4.07 (3.49, 4.69)	3.90 (2.77, 5.76)	25.71 (22.29, 29.17)	13.04 (11.08, 15.29)	13.01 (11.80, 14.30)	13.48 (11.26, 16.51)
6	03.11.-09.11.21	4.98 (4.02, 6.32)	4.87 (3.55, 6.36)	3.94 (3.23, 4.66)	2.97 (2.19, 4.08)	21.46 (19.08, 24.15)	13.84 (11.23, 16.89)	14.84 (13.10, 16.92)	11.68 (9.84, 13.81)
7	17.11.-23.11.21	5.22 (3.67, 7.19)	3.53 (2.56, 4.71)	2.83 (2.41, 3.30)	2.18 (1.63, 2.87)	21.26 (18.45, 24.44)	11.16 (9.32, 13.43)	11.53 (10.30, 12.79)	8.83 (6.40, 11.49)
8	08-12.-17.12.21	3.89 (2.90, 5.13)	4.34 (2.62, 6.75)	3.35 (2.80, 4.02)	3.58 (2.33, 5.40)	18.72 (15.52, 22.70)	12.51 (8.74, 17.72)	13.20 (11.31, 15.37)	13.48 (9.68, 18.58)
9	24-12.-31.12.21	4.32 (3.54, 5.28)	2.95 (2.19, 3.85)	2.95 (2.34, 3.71)	2.44 (2.00, 2.88)	22.02 (18.84, 25.53)	9.07 (7.58, 10.86)	11.28 (9.65, 13.20)	10.10 (8.26, 11.92)
10	14.05.-21.05.20	5.20 (3.65, 7.09)	3.89 (2.33, 5.80)	2.65 (2.15, 3.25)	2.41 (1.99, 2.90)	23.99 (18.80, 30.04)	10.41 (7.70, 13.72)	10.04 (8.53, 11.63)	9.31 (7.50, 11.14)
11	28.05.-04.06.20	5.80 (3.86, 8.42)	3.31 (2.48, 4.39)	3.02 (2.52, 3.57)	2.58 (2.07, 3.41)	19.35 (16.79, 22.09)	10.37 (8.81, 12.25)	11.87 (10.56, 13.27)	10.60 (9.06, 12.45)
12	11.06.-22.06.20	5.04 (3.68, 6.80)	3.03 (2.22, 4.01)	3.02 (2.42, 3.77)	2.13 (1.87, 2.41)	20.36 (17.61, 23.10)	10.39 (8.06, 13.42)	12.62 (10.60, 15.23)	10.18 (8.70, 11.62)
13	26.06.-01.07.20	4.45 (3.47, 5.66)	2.37 (1.81, 3.13)	2.65 (2.20, 3.28)	1.89 (1.64, 2.21)	22.53 (19.00, 26.34)	8.94 (7.47, 10.59)	11.13 (9.78, 12.76)	9.31 (8.04, 10.85)
14	09.07.-16.07.20	4.29 (3.50, 5.22)	2.77 (2.02, 3.73)	2.19 (1.85, 2.59)	2.04 (1.74, 2.42)	20.03 (16.97, 23.59)	7.99 (6.46, 10.03)	8.91 (7.88, 10.14)	9.90 (8.21, 11.76)
15	24.07.-29.07.20	5.12 (3.88, 6.96)	1.97 (1.51, 2.60)	2.27 (1.83, 2.79)	2.31 (1.65, 3.35)	20.83 (16.94, 25.35)	5.72 (4.83, 6.56)	8.26 (7.43, 9.12)	9.51 (7.89, 11.08)
16	07.08.-11.08.20	3.19 (2.34, 4.66)	2.00 (1.48, 2.73)	2.25 (1.85, 2.73)	2.46 (1.90, 3.16)	16.81 (14.35, 19.27)	8.20 (6.94, 9.71)	9.72 (8.34, 11.50)	10.26 (8.48, 12.07)
17	04.09.-09.09.20	3.66 (2.50, 5.50)	2.88 (1.68, 4.48)	1.91 (1.64, 2.21)	1.76 (1.44, 2.10)	17.94 (14.76, 21.50)	8.33 (5.64, 12.00)	8.83 (7.78, 10.10)	8.27 (6.54, 10.10)
18	30.09.-05.10.20	4.13 (3.37, 5.04)	2.34 (1.70, 3.25)	2.01 (1.65, 2.47)	1.64 (1.39, 1.90)	21.64 (18.10, 25.55)	8.54 (6.28, 11.67)	9.46 (7.96, 11.22)	8.28 (6.81, 9.69)
19	14.10.-21.10.20	4.79 (3.62, 6.39)	2.78 (1.82, 4.26)	2.22 (1.75, 2.79)	1.73 (1.41, 2.05)	21.98 (18.64, 25.53)	9.31 (6.49, 13.43)	8.35 (7.38, 9.49)	9.00 (7.53, 10.38)
20	05.11.-10.11.20	3.15 (2.44, 4.08)	2.04 (1.50, 2.75)	2.36 (1.86, 2.97)	1.70 (1.44, 1.97)	17.38 (14.74, 20.27)	6.30 (5.24, 7.55)	9.15 (7.91, 10.43)	9.84 (8.23, 11.65)
21	25.11.-30.11.20	4.96 (4.34, 5.64)	2.83 (2.24, 3.57)	2.60 (2.21, 3.07)	1.80 (1.51, 2.11)	23.51 (20.93, 26.52)	8.69 (7.19, 10.41)	9.01 (8.09, 10.04)	8.17 (7.09, 9.26)
22	09.12.-15.12.20	5.06 (4.22, 6.00)	2.23 (1.91, 2.57)	2.44 (2.03, 2.94)	1.69 (1.48, 1.91)	24.53 (21.26, 28.08)	7.64 (6.74, 8.66)	8.61 (7.91, 9.40)	8.28 (7.38, 9.20)
23	23.12.-30.12.20	5.52 (4.62, 6.60)	2.30 (1.78, 2.89)	2.64 (2.19, 3.14)	2.01 (1.71, 2.35)	26.19 (22.51, 30.25)	7.62 (6.56, 8.73)	9.92 (8.80, 11.15)	8.85 (7.70, 10.04)
24	28.01.-02.02.21	5.32 (4.27, 6.52)	2.76 (2.10, 3.57)	2.95 (2.37, 3.66)	1.82 (1.56, 2.09)	20.72 (18.44, 23.10)	7.90 (6.79, 9.15)	9.62 (8.69, 10.59)	9.20 (7.97, 10.59)
25	24.02.-03.03.21	3.64 (3.09, 4.23)	3.28 (2.62, 4.05)	2.67 (2.31, 3.13)	1.92 (1.59, 2.29)	19.53 (17.18, 21.87)	9.47 (7.83, 11.59)	9.10 (8.37, 9.92)	8.48 (7.49, 9.59)
26	17.03.-26.03.21	5.23 (4.35, 6.22)	2.71 (2.15, 3.35)	2.94 (2.49, 3.50)	2.47 (1.93, 3.13)	24.65 (21.51, 28.25)	8.49 (7.04, 10.17)	9.79 (8.72, 10.98)	8.32 (7.42, 9.40)
27	07.04.-15.04.21	7.37 (6.31, 8.56)	2.69 (2.23, 3.22)	3.08 (2.60, 3.63)	4.08 (2.78, 5.65)	32.80 (28.33, 37.35)	8.69 (7.53, 9.83)	9.81 (8.80, 10.87)	9.51 (8.24, 10.91)
28	12.05.-24.05.21	6.55 (5.59, 7.55)	2.59 (2.13, 3.11)	3.32 (2.64, 4.15)	2.30 (1.89, 2.78)	28.76 (25.21, 32.49)	8.83 (7.50, 10.29)	9.89 (8.66, 11.20)	8.01 (7.14, 8.92)
29	26.05.-03.06.21	5.43 (4.48, 6.57)	3.03 (2.51, 3.61)	2.91 (2.45, 3.49)	2.29 (1.87, 2.83)	25.09 (21.69, 28.75)	9.61 (8.16, 11.16)	9.23 (8.17, 10.38)	8.27 (7.21, 9.41)
30	07.07.-19.07.21	5.60 (4.77, 6.52)	3.12 (2.50, 3.94)	2.63 (2.24, 3.01)	2.04 (1.62, 2.57)	25.84 (22.53, 29.71)	9.40 (7.91, 11.25)	8.92 (7.89, 9.93)	8.40 (7.36, 9.49)
31	04.08.-13.08.21	5.84 (4.68, 7.26)	2.71 (2.22, 3.25)	2.58 (2.12, 3.12)	2.04 (1.62, 2.52)	25.36 (21.80, 28.94)	9.07 (7.72, 10.58)	9.19 (7.75, 11.07)	7.89 (6.44, 10.04)
32	01.09.-14.09.21	5.53 (4.81, 6.36)	2.58 (2.10, 3.19)	2.93 (2.49, 3.43)	2.32 (1.84, 2.92)	26.16 (23.31, 29.34)	8.07 (6.60, 9.90)	8.89 (8.00, 9.89)	6.92 (6.23, 7.70)
33	22.09.-06.10.21	3.03 (2.73, 3.35)	2.25 (1.77, 2.87)	2.66 (2.11, 3.34)	1.82 (1.59, 2.06)	17.98 (16.42, 19.53)	7.67 (6.80, 8.76)	8.59 (7.88, 9.35)	8.87 (7.97, 9.83)
Overall		5.19 (4.99, 5.40)	3.02 (2.88, 3.17)	2.88 (2.79, 2.98)	2.28 (2.18, 2.40)	23.36 (22.71, 23.99)	9.44 (9.10, 9.83)	10.25 (10.04, 10.49)	9.09 (8.85, 9.38)

2. Household size

Figure AF4. Social contact variation stratified by household size between April 2020 and December 2021 in Germany.
(A) Mean number of contacts per person per day; (B) Mean cumulative duration of contacts per person per day (in hours). For comparison, the stringency of the NPIs in Germany (OxCGRT) over the study period is provided in grey shadow on the right y-axis.

The solid line represents the mean number of contacts; the shaded area illustrates the 95% CI obtained using bootstrapping.

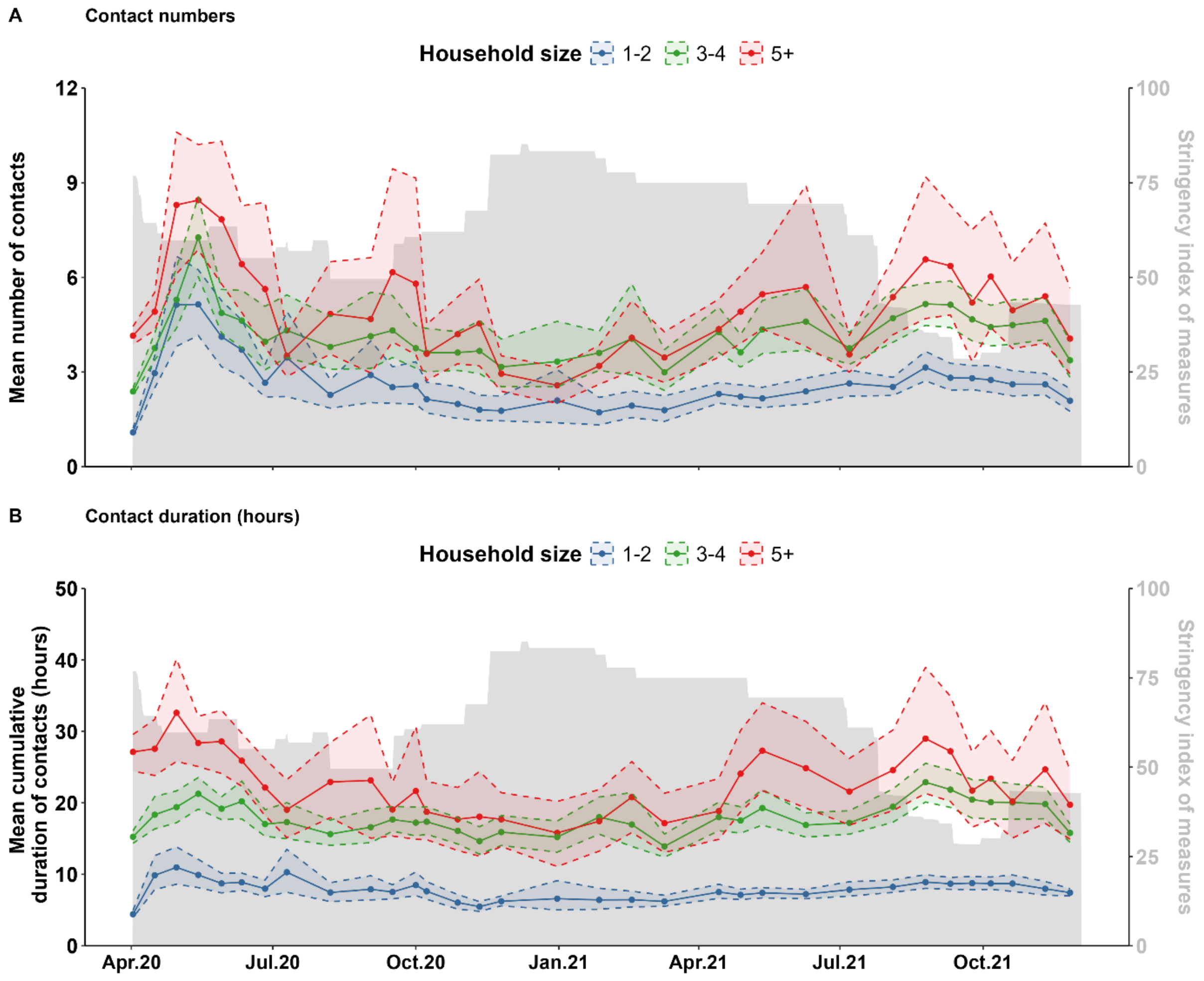

Table AF16. The bootstrapped mean number of contacts and accumulative duration of contacts (hours) with 95%CI across 33 COVIMOD survey waves, stratified by household size.

		Mean number of contacts (95%CI)			Mean duration of contacts in hours (95%CI)		
Wave	Timing	1-2	3-4	5+	1-2	3-4	5+
1	30.04.-06.05.20	1.08 (0.96, 1.21)	2.38 (2.26, 2.50)	4.15 (3.87, 4.44)	4.42 (3.94, 4.96)	15.26 (14.35, 16.09)	27.13 (24.46, 29.57)
2	29.10.-03.11.20	2.96 (2.49, 3.51)	3.76 (3.37, 4.23)	4.91 (4.33, 5.52)	9.86 (7.88, 12.62)	18.34 (16.36, 20.88)	27.58 (23.83, 31.68)
3	09.06.-22.06.21	5.13 (3.81, 6.67)	5.29 (4.38, 6.31)	8.29 (6.11, 10.60)	10.98 (8.62, 13.84)	19.41 (17.22, 21.67)	32.60 (25.80, 40.16)
4	08.10.-20.10.21	5.14 (4.16, 6.24)	7.26 (6.02, 8.55)	8.45 (6.88, 10.21)	9.92 (8.15, 12.10)	21.27 (19.13, 23.54)	28.37 (25.09, 32.11)
5	22.10.-02.11.21	4.12 (3.16, 5.26)	4.87 (4.24, 5.61)	7.84 (5.79, 10.31)	8.73 (7.40, 10.14)	19.19 (17.65, 20.85)	28.60 (24.11, 32.97)
6	03.11.-09.11.21	3.71 (2.86, 4.65)	4.63 (3.76, 5.59)	6.42 (4.89, 8.27)	8.88 (7.76, 10.18)	20.21 (17.77, 23.04)	25.91 (22.33, 29.75)
7	17.11.-23.11.21	2.66 (2.21, 3.23)	3.96 (3.07, 5.08)	5.63 (3.77, 8.37)	8.00 (6.89, 9.20)	17.03 (15.32, 19.05)	22.13 (18.21, 26.15)
8	08-12.-17.12.21	3.46 (2.22, 4.92)	4.32 (3.46, 5.44)	3.51 (2.86, 4.24)	10.30 (7.49, 13.49)	17.30 (14.99, 20.02)	19.04 (15.13, 23.24)
9	24-12.-31.12.21	2.27 (1.85, 2.75)	3.80 (3.08, 4.76)	4.84 (3.57, 6.49)	7.47 (6.19, 8.83)	15.64 (14.06, 17.59)	22.93 (17.93, 28.44)
10	14.05.-21.05.20	2.90 (2.03, 3.99)	4.13 (3.11, 5.53)	4.68 (2.97, 6.62)	7.91 (6.42, 9.80)	16.60 (14.42, 19.05)	23.15 (15.07, 32.32)
11	28.05.-04.06.20	2.52 (2.01, 3.17)	4.32 (3.46, 5.42)	6.16 (3.95, 9.44)	7.53 (6.56, 8.54)	17.68 (16.05, 19.51)	19.07 (15.36, 22.88)
12	11.06.-22.06.20	2.56 (1.98, 3.33)	3.75 (3.12, 4.46)	5.80 (3.55, 9.15)	8.49 (7.03, 10.34)	17.23 (15.38, 19.42)	21.66 (14.91, 30.67)
13	26.06.-01.07.20	2.13 (1.70, 2.67)	3.61 (3.01, 4.38)	3.58 (2.73, 4.51)	7.63 (6.52, 8.97)	17.37 (15.61, 19.46)	18.74 (14.85, 23.00)
14	09.07.-16.07.20	1.99 (1.53, 2.53)	3.62 (3.05, 4.28)	4.20 (3.26, 5.38)	6.05 (5.13, 7.17)	16.08 (14.25, 17.91)	17.70 (13.33, 22.15)
15	24.07.-29.07.20	1.80 (1.46, 2.26)	3.67 (2.95, 4.62)	4.54 (3.19, 5.95)	5.48 (4.83, 6.18)	14.65 (12.82, 16.66)	18.07 (12.55, 24.40)
16	07.08.-11.08.20	1.77 (1.45, 2.24)	3.16 (2.54, 4.04)	2.95 (2.39, 3.52)	6.24 (5.60, 6.97)	15.91 (14.04, 18.22)	17.70 (13.89, 21.43)
17	04.09.-09.09.20	2.09 (1.39, 3.07)	3.33 (2.53, 4.60)	2.58 (2.01, 3.15)	6.60 (5.02, 9.12)	15.21 (13.11, 17.46)	15.80 (11.10, 20.20)
18	30.09.-05.10.20	1.72 (1.32, 2.19)	3.61 (3.05, 4.29)	3.19 (2.61, 3.85)	6.42 (5.12, 8.05)	18.01 (15.63, 20.82)	17.44 (13.25, 21.86)
19	14.10.-21.10.20	1.93 (1.56, 2.40)	4.06 (2.90, 5.80)	4.08 (3.03, 5.26)	6.44 (5.44, 7.64)	16.98 (13.91, 21.44)	20.81 (15.85, 25.77)
20	05.11.-10.11.20	1.79 (1.44, 2.24)	2.99 (2.42, 3.71)	3.46 (2.67, 4.27)	6.22 (5.54, 7.08)	13.91 (12.39, 15.64)	17.16 (13.07, 21.35)
21	25.11.-30.11.20	2.31 (2.01, 2.66)	4.26 (3.62, 5.05)	4.36 (3.47, 5.31)	7.52 (6.64, 8.60)	18.00 (16.11, 20.12)	18.84 (14.91, 23.40)
22	09.12.-15.12.20	2.22 (1.92, 2.59)	3.62 (3.15, 4.18)	4.91 (3.91, 6.05)	7.13 (6.48, 7.92)	17.54 (15.77, 19.46)	24.08 (18.74, 30.14)
23	23.12.-30.12.20	2.16 (1.87, 2.51)	4.35 (3.58, 5.27)	5.46 (4.36, 6.79)	7.41 (6.71, 8.15)	19.28 (16.89, 21.87)	27.31 (21.70, 34.01)
24	28.01.-02.02.21	2.39 (1.98, 2.80)	4.60 (3.69, 5.63)	5.69 (3.81, 8.91)	7.23 (6.58, 7.90)	16.90 (15.21, 18.60)	24.86 (19.29, 31.40)
25	24.02.-03.03.21	2.64 (2.24, 3.06)	3.75 (3.24, 4.25)	3.56 (3.00, 4.16)	7.85 (6.96, 8.97)	17.20 (15.62, 18.91)	21.60 (16.89, 26.22)
26	17.03.-26.03.21	2.53 (2.25, 2.83)	4.71 (3.94, 5.61)	5.37 (4.18, 6.59)	8.23 (7.47, 9.21)	19.46 (17.29, 21.95)	24.57 (18.92, 30.19)
27	07.04.-15.04.21	3.14 (2.72, 3.65)	5.15 (4.48, 5.81)	6.57 (4.69, 9.19)	8.91 (8.04, 9.95)	22.89 (20.13, 25.55)	29.00 (21.32, 38.97)
28	12.05.-24.05.21	2.82 (2.42, 3.28)	5.13 (4.41, 5.89)	6.36 (4.81, 8.29)	8.69 (7.91, 9.57)	21.86 (19.37, 24.56)	27.22 (20.21, 34.92)
29	26.05.-03.06.21	2.80 (2.44, 3.21)	4.67 (3.99, 5.40)	5.20 (3.33, 7.51)	8.77 (7.85, 9.71)	20.46 (17.88, 23.25)	21.72 (16.60, 27.12)
30	07.07.-19.07.21	2.75 (2.36, 3.20)	4.42 (3.83, 5.11)	6.02 (4.40, 8.09)	8.73 (7.90, 9.62)	20.10 (17.76, 23.12)	23.41 (17.64, 30.11)
31	04.08.-13.08.21	2.61 (2.24, 3.04)	4.49 (3.88, 5.29)	4.96 (3.74, 6.46)	8.71 (7.62, 9.91)	20.06 (17.72, 22.68)	20.19 (15.09, 25.95)
32	01.09.-14.09.21	2.61 (2.27, 2.96)	4.62 (4.03, 5.34)	5.40 (3.91, 7.72)	7.98 (7.10, 9.00)	19.83 (17.78, 22.21)	24.69 (17.14, 34.08)
33	22.09.-06.10.21	2.09 (1.75, 2.47)	3.37 (2.83, 3.99)	4.06 (2.94, 5.67)	7.44 (6.96, 7.99)	15.79 (14.48, 17.03)	19.74 (14.92, 24.55)
Overall		2.58 (2.49, 2.67)	4.22 (4.09, 4.36)	5.24 (4.95, 5.56)	7.86 (7.66, 8.06)	18.16 (17.75, 18.53)	23.16 (22.24, 24.09)
